# Orchestrating structure and chemistry dynamics for cluster catalysis

**DOI:** 10.1093/nsr/nwag072

**Published:** 2026-02-04

**Authors:** Jia-Lan Chen, Hong-Yue Wang, Chuan-Liang Ruan, Jin-Xun Liu, Wei-Xue Li

**Affiliations:** State Key Laboratory of Precision and Intelligent Chemistry, University of Science and Technology of China, Hefei 230026, China; Department of Chemical Physics, School of Chemistry and Materials Science, University of Science and Technology of China, Hefei 230026, China; State Key Laboratory of Precision and Intelligent Chemistry, University of Science and Technology of China, Hefei 230026, China; Department of Chemical Physics, School of Chemistry and Materials Science, University of Science and Technology of China, Hefei 230026, China; State Key Laboratory of Precision and Intelligent Chemistry, University of Science and Technology of China, Hefei 230026, China; Department of Chemical Physics, School of Chemistry and Materials Science, University of Science and Technology of China, Hefei 230026, China; State Key Laboratory of Precision and Intelligent Chemistry, University of Science and Technology of China, Hefei 230026, China; Department of Chemical Physics, School of Chemistry and Materials Science, University of Science and Technology of China, Hefei 230026, China; Hefei National Laboratory, University of Science and Technology of China, Hefei 230088, China; State Key Laboratory of Precision and Intelligent Chemistry, University of Science and Technology of China, Hefei 230026, China; Department of Chemical Physics, School of Chemistry and Materials Science, University of Science and Technology of China, Hefei 230026, China; Hefei National Laboratory, University of Science and Technology of China, Hefei 230088, China

**Keywords:** theoretical catalysis, dynamics of cluster catalysis, supported metal clusters, structure and chemistry dynamics

## Abstract

Supported metal clusters maximize atom efficiency and expose diverse low-coordination metal motifs, but under reaction conditions, they are inherently fluxional—adsorbed reactants can constantly reform and even break the underlying metal–metal and metal–support bonds, generating an ensemble of metastable structures for catalysis. Identification of the interplay between supported clusters and surface chemistries is vital but a challenge for their complex dynamic evolutions. Here, we uncover three characteristic and universal regimes: (i) a fluxional regime, where fast restructuring erases site individuality; (ii) a kinetically trapped regime, where slow restructuring freezes the catalyst into a single geometry; and (iii) a unique coupled regime, where structural dynamics and chemistry occur on comparable timescales and where multiple metastable motifs actively participate in turnover. Moreover, we identify a single, dimensionless metric, *N*_c_ *=* *τ*_struct_*/τ*_chem_, the ratio between the structural rearrangement timescale (*τ*_struct_) and the chemical residence time of the reactant (*τ*_chem_), to differentiate these three regimes with distinct activity and stability. It is found that *N*_c_ should be neither too small (fluxional regime) nor too large (kinetically trapped regime). When the optimal value *N*_c_ ∼ 1 is approached (coupled regime), structural and chemical ‘clocks’ match, enabling the multiple active metastable isomers to persist long enough to participate in turnover and maximize reaction rates. Using CO adsorption–desorption on size-selected Cu*_n_*/TiO_2_(110) clusters as a model system, we demonstrate a master kinetic curve versus *N*_c_ and reveal tunable levers that drive clusters into the optimal coupled regime. Trends generalize across metals: coinage clusters (Ag, Au) prefer fluxionality, Rh/Pd favor trapping, and Cu and Pt/Ru often lie near the coupled boundary. Time-scale matching thus emerges as a design rule for adaptive, fluxional catalysts with high activity and stability at the same time.

## Teaser

This work shows that catalysis on supported metal clusters is governed by the time-scale ratio NC=τstruct/τchem, and that matching structural rearrangement and reaction times (NC≈1) defines a coupled regime in which metastable isomers become active sites and catalytic activity is maximized.

## INTRODUCTION

Supported metal clusters occupy the size regime between single atoms and nanoparticles, combining near-maximal metal utilization with a dense variety of low-coordination motifs [1–9]. The electronic structure and reactivity of these materials are strongly shaped by the support through charge transfer, strain and anchoring, which in turn tune the adsorption and reaction pathways [10–15]. These attributes make clusters attractive for transformations central to sustainable chemistry, including CO_2_ conversion and selective hydrogenation [16–23]. A defining feature of clusters under reaction conditions is fluxionality [24–31]; adsorbates continually break and reform metal–metal and metal–support bonds so that catalysis proceeds over an evolving ensemble rather than a single static site [32–38]. In this regime, the structure and chemistry coevolve; transient configurations can stabilize key intermediates or open pathways invisible to static pictures [39,40].

Despite increasing *operando* evidence—coordination–number oscillations, reversible disordering and broadened vibrational bands—the field lacks a general rule for when structural dynamics control rates and how to tune them. Present models typically assume either a fixed structure or instantaneous equilibration of the cluster ensemble [41–45], offering no quantitative language to distinguish ‘productive dynamics’ from ‘detrimental disorders’. The core difficulty is a time-scale mismatch: cluster restructuring follows networks of isomerizations with distributed barriers and entropic weights [27,46–48], whereas adsorption, desorption and bond-forming steps evolve on chemical clocks set by binding energetics and reaction barriers [49–56]. These processes often operate on comparable timescales under working conditions, precisely where decoupled assumptions fail and where history-dependent rates, non-Arrhenius kinetics, and coverage- or defect-controlled selectivity emerge [57–62]. As a result, catalyst design remains empirical, and the lack of a predictive theory for fluxional clusters is now a major bottleneck for advancing atom-efficient catalysis.

Here, we discover that cluster catalysis is governed by three universal dynamical regimes: (i) a fluxional regime where fast restructuring averages out site individuality; (ii) a kinetically trapped regime where slow restructuring freezes a single geometry during turnover; and (iii) a previously unrecognized coupled regime where structural rearrangements and surface chemistry proceed on comparable time scales so that multiple metastable motifs actively carry out the reaction. We introduce a single, dimensionless control parameter, *N*_c_ *=* *τ*_struct_*/τ*_chem_, the ratio of the structural relaxation time to the chemical residence time, to diagnose these regimes with distinct reactivity and stability. We found that *N*_c_ should be neither too small (fluxional regime) nor too large (kinetically trapped regime). When the optimal *N*_c_ ∼ 1 is approached (coupled regime), maximized activity along with excellent stability arises, where the structural and chemical clocks match.

We establish and test this framework for CO adsorption–desorption over size-selected Cu*_n_*/TiO_2_(110) clusters (*n* = 2–10), a model step relevant to CO_2_ hydrogenation. First-principles energetics combined with kinetic-network simulations collapse the near-thermodynamic ensemble desorption rates onto a single master curve versus *N*_c_, revealing a non-monotonic size effect that originates from dynamical coupling. We further identify actionable levers—composition and size (landscape connectivity), coverage (*τ*_chem_) and support defect density (*τ*_struct_)—that drive catalysts into the optimal coupled window. In the fluxional limit (*N*_c_ ≪ 1), structures re-equilibrate too quickly for transient active motifs to persist, yielding negligible gains. In the trapped regime (*N*_c_ ≫ 1), sluggish restructuring strands the catalyst in long-lived, less-active states that hinder turnover, whereas in the coupled window (*N*_c_ ∼ 1) rearrangement is agile yet slow enough for low-barrier sites to survive through turnover and maximize rates. Trends generalize across metals; coinage clusters prefer fluxionality, Rh/Pd favor trapping, and Cu and Pt/Ru often reside near the coupled boundary. Together, these results elevate time-scale matching to a design principle for adaptive, fluxional catalysts—providing a quantitative, experimentally testable route to stabilize reactive non-ground-state configurations during turnover and to move beyond empirical optimization.

## RESULTS AND DISCUSSION

### Derivation of the *N*_c_ concept and its implications for catalytic activity

We first develop a quantitative framework that links surface reconstruction and chemistry through two clocks: the structural relaxation time (*τ*_struct_) and the chemical residence/turnover time (*τ*_chem_). Gas-surface adsorption/desorption rates are obtained from collision theory, whereas elementary reconstruction and reaction rates follow transition‐state theory (TST). The resulting rate set is propagated with kinetic Monte Carlo (KMC) (Notes S1–2, Fig. S1). To compute *τ*_struct_, we enumerate cluster isomers, calculate all isomer barriers (*E*_a,recon_), and assemble a transition network. The KMC simulation is initialized from a randomized isomer distribution—mimicking kinetically trapped, atomically precise subnanoclusters on a support—and runs to equilibrium. The time required for the ensemble to reach its Boltzmann distribution defines *τ*_struct_ and yields steady-state isomer populations. To obtain *τ*_chem_, we extend the network to include adsorbed and reactive states by computing barriers for the key chemical steps (adsorption, desorption and surface reactions). The joint structural–chemical network is then evolved by the KMC to a steady state, and residence/turnover statistics provide *τ*_chem_.

We define their ratio, *N*_c_ = *τ*_struct_/*τ*_chem_, as a dimensionless cycle count that quantifies the dynamic coupling between surface reconstruction and chemical turnover. Depending on *N*_c_, we anticipate three distinct kinetic regimes in cluster catalysis:

- Thermodynamic ensemble regime (*N*_c_ ≪ 1) under τ_struct_ ≪ *τ*_chem_. In this fluxional limit, rapid restructuring erases site individuality. Between turnovers, the cluster re-equilibrates to a near-thermodynamic ensemble, so the measured rate is an average over many accessible configurations (near-thermodynamic ensemble catalysis). Overall performance is limited because continual reconfiguration prevents sustained occupancy of the most active isomer under reaction conditions.
- Kinetically trapped regime (*N*_c_ ≫ 1) under τ_struct_ ≫ *τ*_chem_. The catalyst structure remains rigid in a specific yet metastable state over multiple reaction cycles, as it is not able to rearrange itself to favorable one within the timescale of the turnover. Consequently, reactivity is governed by this specific configuration. Therefore, the reaction rate is primarily determined by the residence time of reactants or intermediates on the rigid catalyst surface.
- Coupled regime (*N*_c_ ∼ 1) under *τ*_struct_ ∼ *τ*_chem_. Structural reconfiguration and catalytic events occur on comparable timescales, with the two processes effectively synchronized. In this unique regime, metastable isomers, which would typically be too short-lived, can persist as long as the chemical reaction, often becoming the true active species for catalysis. The catalyst rarely resides in its global minimum structure; instead, higher-energy metastable configurations remain operational for significant portions of the reaction cycle. This dynamic interaction enhances catalytic efficiency by allowing the catalyst to adapt to reaction conditions while maintaining optimal turnover rates.

**Figure 1.**
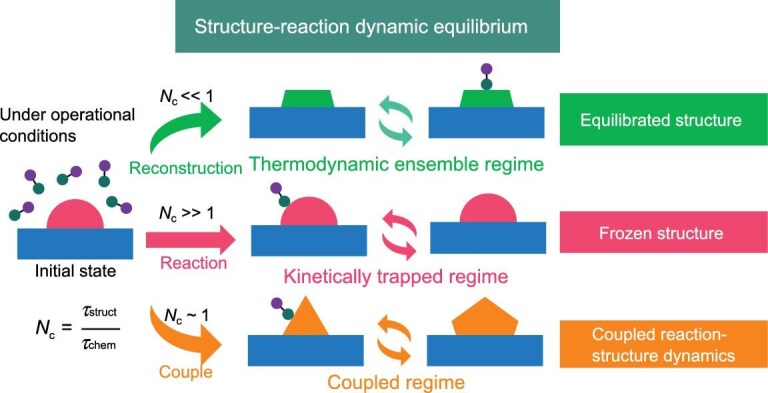
Schematic illustration of the dynamic coupling between structural fluxionality and catalytic turnover. Supported nanoclusters undergo different regimes on the basis of the time scale comparison between structural relaxation (*τ*_struct_) and catalytic turnover (*τ*_chem_). For *N*_c_ ≪ 1, structural reconstruction occurs before the reaction, leading to a sequence of metastable state transitions before reaching the fixed stable state, where the reaction proceeds. In contrast, when *N*_c_ ≫ 1, the structure remains fixed, and the reaction occurs at quasiequilibrium. When *N*_c_ ∼ 1, the system reaches a structure‒reaction coupled process, where structural dynamics and catalytic activity are mutually influenced.

This concept is illustrated schematically in Fig. 1. During turnover, a cluster hops among isomers. When the structural clock matches (*τ*_struct_ ∼ *τ*_chem_) or lags the chemical clock (*τ*_struct_ > *τ*_chem_), each metastable isomer can complete one or several turnovers before relaxing and thus becomes an operationally active state. The catalyst design therefore reduces to tuning the cycle count *N*_c_ = *τ*_struct_/*τ*_chem_—via temperature, coverage and support interactions—to either harness or suppress these transient states. Because structure and chemistry are bidirectionally coupled, longer adsorbate residence (larger *τ*_chem_) affords more time for rearrangement, whereas ongoing reconstruction reshapes sites and barriers, feeding back to *τ*_chem_. In short, reactant lifetimes set the window for restructuring, and restructuring rewrites those lifetimes.

### Dynamic coupling in Cu cluster catalysts

**Figure 2.**
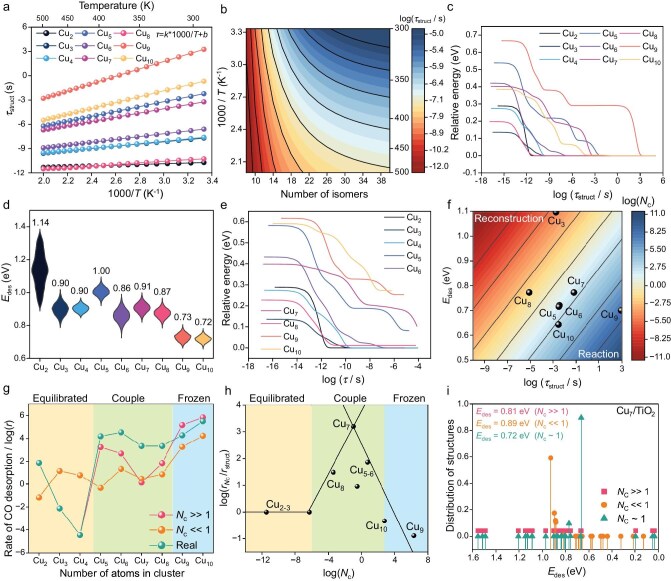
Dynamic coupling in Cu*_n_*/TiO_2_ (*n* = 2–10) clusters under CO. (a) Temperature-dependent structural equilibration time (*τ*_struct_) over the Cu_2_‒Cu_10_ clusters between 300 and 500 K. (b) 2D phase diagram of *τ*_struct_ as a function of the number of isomers and temperature, revealing the exponential growth of relaxation time with increasing structural complexity. (c) Time-dependent evolution of the relative energy landscape for structural reconstruction at 300 K, indicating that larger clusters exhibit deeper potential wells and slower relaxation. (d) Violin plots of the CO desorption energies (*E*_des_) of CO over the Cu_2_‒Cu_10_ clusters. The width of each violin represents the statistical distribution of adsorption sites and configurations, revealing that smaller clusters (Cu_2_–Cu_5_) present greater and broader *E*_des_ distributions owing to stronger and more heterogeneous CO binding, whereas larger clusters (Cu_6_–Cu_10_) present narrower, weaker adsorption characteristics associated with surface delocalization. (e) Relative energy trajectories during CO adsorption–desorption dynamics at 300 K, showing a gradual approach toward equilibrium with characteristic relaxation plateaus. (f) 2D phase diagram of the coupling time until the thermodynamic equilibrium of the structure and reaction couple is reached when reconstruction and CO equilibrium at 300 K are considered. (g) Comparison of CO adsorption–desorption rates across the three dynamical regimes: the fluxional regime (*N*_c_ ≪ 1, relaxed structure ensemble), the kinetically trapped regime (*N*_c_ ≫ 1, frozen structure) and the practically relevant regime. (h) Scatter plot of *r_Nc_*/*r*_struct_ as a function of *N*_c_. Here, *r*_Nc_ is the microkinetic rate evaluated with finite time-scale coupling, and *r*_struct_ is the equilibrium structure reference rate. (i) Comparison of CO desorption energies distribution (*E*_des_) across the three regimes at Cu_7_/TiO_2_. The lowest effective barrier (0.72 eV) occurs at *N*_c_ ∼ 1, corresponding to the dynamically coupled regime, demonstrating that synchronization between reconstruction and reaction maximizes catalytic activity.

We first quantified the adsorbate-free structural dynamics of the Cu*_n_* (*n* = 2–10) clusters on TiO_2_(110) (Figs S2 and S3). From density functional theory (DFT)-derived isomerization networks, we computed the structural equilibration time, *τ*_struct_, defined as the time for a cluster initialized in a high-energy configuration to relax to its Boltzmann ensemble at a given temperature (Fig. 2a, Note S3, Table S1). This thermodynamic equilibrium time characterizes how quickly the system reaches its stable surface configuration after perturbation, which is crucial for understanding the catalytic process dynamics. Across 300–500 K of Cu_2–8_ clusters, *τ*_struct_ spans >6 orders of magnitude (∼10^−12^–10^−6^ s). Cu_9_ is markedly sluggish (∼10^−3^–10^3^ s), and the remaining medium Cu_10_ falls between ∼10^−6^–10^−2^ s. To assess finite-temperature effects, 100 ps neural network potential molecular dynamics NNP-MD at 500 K [reverse water-gas shift (RWGS)-relevant] was employed. Cu_5_ explores four metastable basins before re-equilibrating, yet the 0 K global minimum remains dominant; despite broader distributions from entropy, the energetic ordering and kinetics are unchanged. Accordingly, the size-dependent *N*_c_ trends and rate gains *G* are robust to finite-temperature corrections (Figs S4 and S5). Moreover, NNP-MD simulations (500 K, 100 ps) for representative Cu_5_ clusters map the free-energy landscape and barrier statistics; finite-*T* sampling preserves the static-DFT hierarchy and transition barriers within uncertainty, supporting the use of 0 K energetics for *N*_c_ and *G*. This pronounced, non-monotonic size dependence indicates that small atomic changes can radically alter fluxionality.

The origin of this trend is the topology and connectivity of each cluster’s energy landscape (Table S1). Cu_2–8_ features many low-energy isomers connected by low activation barriers (∼0.27‒0.52 eV), enabling rapid interconversion. In contrast, Cu_9_, although rich in isomers (42 within the relevant window), exhibits higher median activation barriers and geometric constraints that also slow relaxation (∼0.89 eV). Similarly, Cu_10_ presents very few basins separated by high activation barriers (∼0.70 eV), leading to long-lived kinetic trapping. This suggests a design rule that maximizes network connectivity while limiting excessive structural degeneracy but with low transformation activation barriers to obtain fluxional yet controllable clusters.

We compared cluster landscapes on a 2D descriptor map (Fig. 2b) that relates isomer count and network connectivity (Note S3, Fig. S6) to temperature. Complex landscapes are highly temperature sensitive; as additional pathways are activated, *τ*_struct_ can decrease from ∼10^−5^ s at 300 K to ∼10^−8^ s at 500 K when the number of isomers is greater than 10. In contrast, simple landscapes are fast, almost regardless of temperature, maintaining *τ*_struct_ of ∼10^−10^–10^−12^ s across the same range when the number of isomers is less than 15. The relaxation trajectories (Fig. 2c) echo this contrast. At 300 K, Cu_2_ and Cu_8_ quickly funnel into isomers near the ground state, whereas Cu_9_ shows a bimodal energy–time profile—lingering in a high-energy basin before a delayed drop to the low-energy manifold (>10^3^ s). Most other sizes (*τ*_struct_ ∼10^−9^‒10^−2^ s) traverse several metastable states 0.10–0.40 eV above the minimum route to equilibrium. Therefore, richly connected landscapes accelerate sampling but also harbor deep kinetic traps, jointly shaping *operando* behavior. To justify the assumption of a fixed cluster size, we evaluated the energetics of fragmentation and sintering via nudged elastic band (NEB) and NNP-MD simulations (Fig. S7). The high fragmentation barrier (2.17 eV for Cu_10_ → Cu_9_ + Cu_1_) precludes Ostwald ripening. Furthermore, while NNP-MD simulations at 500 K identify particle-mediated migration and coalescence (PMC) as the potential sintering mechanism, the low diffusion coefficient (10^−5^^–^10^−7^ cm^2^/s) and the experimental use of low metal loadings ensure that sintering is kinetically suppressed. Bifunctional heteroenergetic supports [8]—combining strong and weak metal–support interactions (MSIs), such as TiO_2_ and ZrO_2_/MgO—effectively stabilize clusters and suppress the PMC ripening processes. Therefore, the structural integrity of the clusters is maintained on the timescale of the catalytic reaction.

We next assessed the chemical clock, *τ*_chem_, for CO adsorption–desorption over Cu_2_–Cu_10_ via DFT binding energies and microkinetic analysis through collision theory (Fig. 2d, Tables S2 and S3). This choice is predicated on the dual role of CO as both a reactant and a structural modifier [63]. Many transition metal-catalyzed reactions, such as CO oxidation [64], the RWGS [65] and the water–gas shift (WGS) [66] or steam reforming of methane (SRM) [10], CO desorption or surface residence is the rate-limiting step that competes with further elementary steps (e.g. dissociation or hydrogenation). Our current investigation focuses specifically on the fundamental synchronization between structural fluxionality and the adsorption/desorption cycle; however, establishing the comprehensive link between this timescale and specific reaction coordinates remains a primary objective for our future research. As the benchmark, the adsorption/desorption mechanism was validated via enhanced sampling simulations using an NNP at 500 K (Fig. S8). The results indicate that CO adsorption is nearly barrierless (*E*_a,chem_ < 0.11 eV), justifying the use of *E*_des_ as the sole kinetic barrier. The excellent agreement between the dynamic and static energies (<0.1 eV) ensures the reliability of the thermodynamic binding strength as a proxy for the kinetic bottleneck in our model. From Cu_2_ to Cu_10_, *τ*_chem_ contracts from 7 × 10^2^ s to 9 × 10^−5^ s (∼8 orders of magnitude at 300 K), which is consistent with the decrease in *E*_des_ (Fig. 2d), and this compression of the chemical clock increases *N*_c_, which explains the observed regime shift and activity trend (Table S3), indicating weaker CO binding on larger clusters (Table S2). The general trend is clearly that larger clusters bind CO more weakly, leading to faster desorption and shorter residence times.

With *τ*_struct_ and *τ*_chem_ in hand, we compared their magnitudes across Cu*_n_* to assess dynamic coupling at 300 K (Fig. 2e). For Cu_2_, Cu_8_ and Cu_10_, *τ*_struct_ ∼10^−11^–10^−10^ s ≤ *τ*_chem_, yielding *N*_c_ ≪ 1. These clusters thus re-equilibrate between turnovers, and their activity reflects an ensemble average over isomers. In contrast, Cu_3‒7_ exhibit *τ*_struct_ values of ∼10^−5^–10^−1^ s, which are comparable to their CO residence times, resulting in *N*_c_ ∼ 1. Moreover, Cu_9_ has a *τ*_struct_ value of ∼ 10^3^ s, which is longer than its CO residence time, resulting in *N*_c_ ≫ 1. These clusters cannot fully relax before the next event and therefore turn over on long-lived metastable configurations; under *operando* conditions, the ground state contributes little. Plotting *τ*_struct_ against *τ*_chem_ defines a dynamic phase map (Fig. 2f) where points below the diagonal (*N*_c_ ≪ 1) are fluxional, those above are trapped (*N*_c_ ≫ 1), and those near the diagonal are coupled (*N*_c_ ∼ 1). Specifically, Cu_9_ lies deep in the structure kinetic trap region; Cu_2_, Cu_8_ and Cu_10_ lie in the flux region; and Cu_3–7_ clusters near the coupled boundary.

Here we quantify structure–reaction coupling in CO desorption using the cycle count *N*_c_ (Fig. 2g, Fig. S9 and Table S4). Comparing structural equilibration times (*τ*_struct_) with turnover times (*τ*_chem_) reveals a clear optimum: rates are maximized when *N*_c_ ∼ 1, i.e. when reconstruction and chemistry run on comparable clocks. Relative to the fluxional limit (*N*_c_ ≪ 1) and the trapped limit (*N*_c_ ≫ 1), coupled Cu_5_–Cu_8_ clusters exhibit >2-order increases in the near-thermodynamic ensemble desorption rate under realistic conditions, consistent with a redistribution of population toward highly reactive metastable microstates. In this window, structural flexibility is sufficient to access low-barrier sites, yet slow enough that those sites persist during turnover, enabling efficient CO release.

To quantify this effect, we define the relative rate gain, *G* = *r_Nc_*/*r*_struct_, where *r_Nc_* is the near-thermodynamic ensemble rate from the coupled structural–chemical network and *r*_struct_ is the reference rate evaluated on equilibrated structures (Fig. 2h). On Cu*_n_*/TiO_2_, *G* shows a universal, piecewise dependence on *N*_c_: *G ∼* 1 for *N*_c_ < 10^−4^ (fast-structure, quasi-equilibrium); *G* rises sharply and peaks as *N*_c_ = 1 (coupled window); and for *N*_c_ ≫ 1 it returns toward unity (reaction-gated plateau). A dynamic phase map (Fig. 2i) illustrates the mechanism: at *N*_c_ ∼ 1, Cu_7_/TiO_2_ exhibits a higher density of active sites with lower effective CO desorption energies (∼0.72 eV) than in the fluxional or trapped regimes (∼0.81–0.89 eV).

Size tunes activity chiefly by shifting *N*_c_. In the fluxional limit (*N*_c_ ≪ 1), structures re-equilibrate too quickly for transient active motifs to persist, so small clusters (Cu_2_‒Cu_4_, *N*_c_ < 10^−4^) show negligible gains. By contrast, in the trapped regime (*N*_c_ ≫ 1), sluggish restructuring strands the catalyst in long-lived, less active states, hindering turnover. In the coupled window (*N*_c_ ∼ 1), rearrangement is agile yet slow enough for low-barrier sites to survive through turnover, enabling efficient CO release; mid-sizes (Cu_5_‒Cu_8_) therefore display pronounced enhancement. Consequently, the largest clusters, which relax more slowly than the reaction (*N*_c_ > 10^4^), exhibit diminished rates. Moreover, orthogonal benchmarks behave as expected: the pressure (*P*)–*E*_des_ map shows monotonic rate increases with higher *P* and lower *E*_des_ (Fig. S10)—while *N*_c_ governs the multiplicative gain *G*. Together, these results establish *N*_c_ as a transferable descriptor linking size-dependent dynamics to measurable rate augmentation.

### Influence of metal identity on dynamic regimes

**Figure 3.**
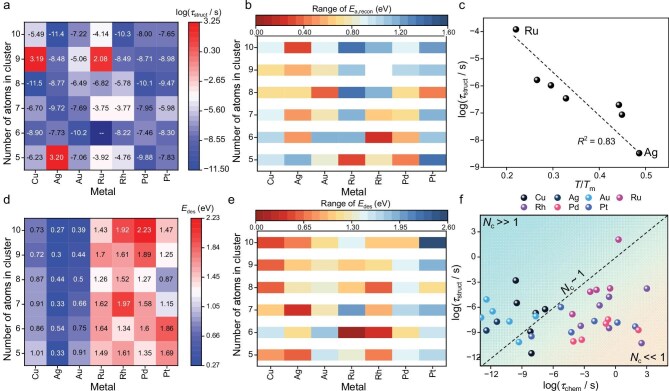
Coupled kinetics under CO equilibrium (a) Reaching structure thermodynamic equilibrium time (*τ*_struct_) over different metals and numbers of atoms in clusters above 300 K. (b) Range of reconstruction forward barrier energies (*E*_a,recon_) over different metals and numbers of atoms in clusters. (c) Relationships between the ratio of temperature to Tammann temperature (*T*/*T*_m_) and the average structure thermodynamic equilibrium time *τ*_struct_ over different metals. (d) CO desorption energies over different metals and the number of atoms in clusters above 300 K. (e) Range of CO desorption energies over different metals and the number of atoms in clusters. (f) 2D phase diagram of the coupling time until the thermodynamic equilibrium of the structure and reaction couple is reached when reconstruction and CO equilibrium at 300 K are considered.

Using the same workflow, we evaluated *τ*_struct_ for 5–10 atom clusters of Au, Ag, Cu, Pt, Pd, Rh and Ru on TiO_2_(110) through isomerization networks (Fig. 3a, Figs S11–S18 and Table S5). Clear periodic trends emerge. Noble metals such as Pt, Pd and Au exhibit relatively rigid and electronically stabilized frameworks, which results in slower and less pronounced structural rearrangements during the equilibration process. This is reflected in their equilibrium times, ranging from 10^−10^ to 10^−5^ s, with Pd and Pt clusters showing the shortest equilibration times at various sizes. This allows them to rapidly adapt and reorganize at the atomic level, promoting faster dynamic coupling. Ru and Rh have the longest equilibrium times (more than 10^−5^ s), except for Rh_6_ and Rh_10_. In contrast, coinage metals such as Cu and Ag demonstrate a greater degree of flux and more pronounced size-dependent structural dynamics (10^−11^ to 10^3^ s). These metals tend to show a greater propensity for metastable configurations. For example, Cu clusters consistently reach thermodynamic equilibrium in the shortest times (Cu_8_: 10^−11^ s) and longest times (Cu_9_: 10^3^ s), underscoring their enhanced flexibility and responsiveness to reactive environments. These metals have greater structural flexibility, particularly in larger clusters.

The distributions of the isomerization activation barriers Δ*E*_recon_ (Fig. 3b and Table S6) cleanly separate the metals. The Cu clusters have narrow spans (<1.0 eV), making most rearrangements thermally accessible near 300 K. Rh and Ru exhibit broad ranges up to more than 1.0 eV, except for Ru_5_ and Rh_7_, implying very slow pathways and long-lived metastable states. Pt is an intermediate (∼1.1–1.3 eV). These trends track bonding ‘softness’: coinage metals (Cu, Ag and Au) with more malleable bonds enable facile interconversion, whereas late transition metals (Rh and Ru) form rigid networks that increase kinetic barriers and favor trapping. A consistent underpinning is their higher Tammann temperatures (*T*_m_)—reflecting stronger cohesive bonding—which correlates with stiffer landscapes and larger Δ*E*_recon_ in the Rh/Ru series than in the coinage series.

The ultrafast dynamics observed in coinage metals can be attributed to their relatively low *T*_m_. For coinage metals, this low value indicates that even at moderate temperature (∼700 K), a considerable fraction of the atomic mobility related to melting becomes activated. As a result, the atoms in these metals can easily rearrange, facilitating rapid structural changes. This high degree of atomic mobility at a high ratio of temperature (300 K) and *T*_m_ is crucial for understanding the behavior of coinage metals in catalytic processes, where atom rearrangement plays a vital role in the reaction kinetics. However, noble Rh and Ru clusters restructure more slowly (several cases with *τ*_struct_ > 10^−5^ s), whereas Pt/Pd clusters are intermediate (10^−8^–10^−5^ s).

The number of accessible isomers scales inversely with the *T*_m_ (Fig. 3c, Note S5, Fig. 19 and Table S7). The structural relaxation time (*τ*_struct_) over different metals clearly inversely correlates with the normalized temperature (*T*/*T*_m_), following an approximately exponential decay trend. Metals with lower *T*/*T*_m_ ratios, such as Ru, Rh and Pt, display sluggish structural dynamics, reflecting stronger bonding rigidity and higher cohesive energy, whereas those approaching higher *T*/*T*_m_ values, such as Cu, Ag and Au, undergo rapid structural fluctuations consistent with increased atomic mobility near the melting regime. Moreover, low-*T*_m_ metals (Cu, Ag) exhibit many isomers and highly connected transition networks, indicative of high atomic mobility and configurational entropy. High-*T*_m_ metal (Rh, Ru) samples have far fewer configurations, reflecting intrinsically rigid landscapes. This confirms that *T*_m_ is a useful proxy for a metal’s atomic mobility and configurational entropy; low-*T*_m_ metals inherently explore broader configuration spaces, whereas high-*T*_m_ metals are confined to narrower ensembles. We evaluated the relationship between cluster cohesive energy and structural reconstruction timescales (*τ*_struct_). As shown in Fig. S20, *τ*_struct_ exhibits a compelling linear dependency on the cohesive energy (*R*^2^ = 0.88), which aligns with the trends observed for the reduced *T*_m_. This correlation remains robust for single-atom alloy (SAA) clusters, such as Cu_4_M_1_/TiO_2_ (Fig. S21), where the cohesive energy of the alloyed system dictates the kinetic barriers for structural rearrangement. These findings demonstrate that cohesive energy is a reliable and universal descriptor for assessing the synchronization between structural and chemical clocks across both monometallic and alloyed subnanometer catalysts. These intrinsic metal-specific traits strongly influence each system’s propensity for dynamic coupling.

CO binding sets the chemical clock. Metal identity governs *τ*_chem_ through CO adsorption strength (Fig. 3d and e, Figs S22 and S23, Tables S8 and S9). Pt/Pd/Rh strongly bind CO (*E*_des_ > 1.3 eV; e.g. Pt_5_ = 1.49 eV, Rh_5_ = 1.67 eV), yielding long residence times (*τ*_chem_ > 10^5^ s) that can electronically pin surface geometries at 300 K. Such strong bonding means that CO molecules linger on the surface (long *τ*_chem_) and can even ‘lock’ the surface structure, as the adsorbed CO imposes an energetically favorable geometry. Ag/Au bind weakly (∼0.3–0.4 eV), resulting in a short *τ*_chem_ (∼10^−11^ s); Cu is an intermediate (∼0.7–1.0 eV and ∼10^−15^–1 s of *τ*_chem_, size dependent). Consequently, strongly bound metals are reaction-limited and tend toward trapped dynamics, whereas weakly bound metals are structure-limited and tend toward the fluxional regime. Notably, strong adsorbate binding can also suppress restructuring (affecting *τ*_struct_), so the operative behavior follows from the joint balance of *τ*_struct_ and *τ*_chem_—conveniently summarized by *N*_c_ = *τ*_struct_/*τ*_chem_.

By jointly considering *τ*_struct_ and *τ*_chem_, we place metals on an *N*_c_ phase map (Fig. 3f and Table S10). The optimal catalysts are those with *N*_c_ ∼ 1, which indicates a balanced coupling between structural dynamics and reaction processes. This avoids excessive structural rearrangement, which could compromise stability and efficiency. Coinage clusters (Ag and Au) restructure rapidly and release CO readily, yielding *N*_c_ ≪ 1 (fluxional regime). Pd and Rh restructure slowly and bind CO strongly, producing *N*_c_ ≫ 1 (kinetically trapped). Cu, Pt and Ru frequently lie near *N*_c_ ∼ 1, indicating intrinsic synchronization between restructuring and turnover, which is consistent with the adaptive behavior observed *operando*. Therefore, metal identity presets the dynamic baseline: reconstruction-dominated (Ag/Au), reaction-dominated (Pd/Rh) or coupled (Cu/Pt/Ru). Among these, the most effective metals for catalytic activity are Cu, which consistently has median *N*_c_ values across various cluster sizes, particularly for clusters in the 5–8 atom range, and Ru/Pt clusters in the 7–10 atom ranges. Thus, selecting the composition is a primary lever for targeting the desired dynamic state.

Extending the analysis across metals (Fig. S24) shows that activity is modulated primarily by shifts in *N*_c_. Ag and Au generally sit in the small *N*_c_ band—fast restructuring and/or weak CO binding—so the relative rate gain *G* remains near unity (little coupling advantage). Rh and Pd, with slower restructuring and stronger binding, populate the large-*N*_c_ band, where structure gating limits turnover and *G* again plateaus. Cu, Ru and Pt most often occupy an intermediate-*N*_c_ window, consistent with partial time-scale matching and modest enhancement. Within each metal family, particle size further tunes *N*_c_ by reshaping barrier networks and *τ*_struct_; sizes that move the system toward *N*_c_ ∼ 1 show the largest *G*, whereas sizes that push *N*_c_ far below or above unity revert to fluxional averaging or kinetic trapping. Thus, metal identity sets the baseline *N*_c_, and size selects where each catalyst lands on the universal *G*(*N*_c_) curve, with maximal gains achieved only near the coupled regime.

### Effects of adsorbate coverage and support on dynamics

**Figure 4.**
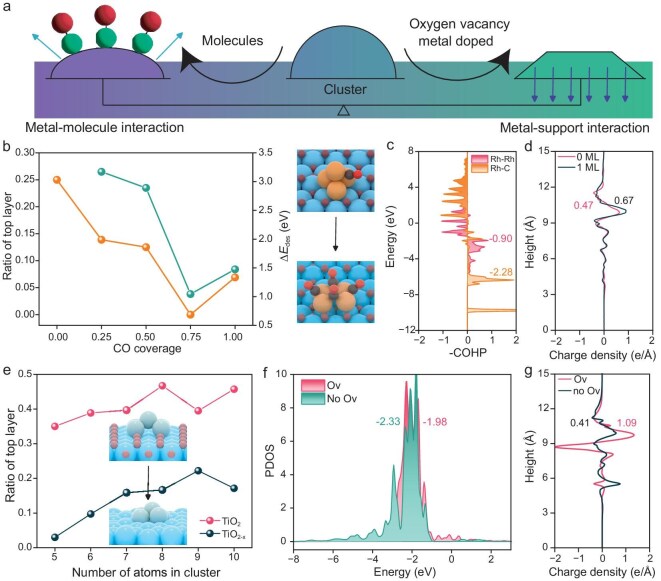
Design to maintain metastable structures. (a) Illustration of controlling metal‒molecule interactions and MSIs. (b) Changes in the ratio of the top layer with increasing CO monolayers. The yellow and green lines represent the ratios of the top layer and desorption energies over different coverages from 0 to 1. The yellow, blue, dark gray, brown and red spheres represent Rh, Ti, C, lattice O, and the O atom in CO, respectively. (c) COHP analysis of the bonds of Rh‒Rh and Rh‒C. The values are integrated COHP (ICOHP) values integrated from −12 to −8. (d) Transfer of charge from the metal to support over 0 and 1 monolayers of CO. (e) Changes in the ratio of the top layer with increasing occurrence of oxygen vacancies. The light blue spheres represent the Cu atoms. (f) *d* orbital of Cu over oxygen vacancies (Ov) and no oxygen vacancies (No Ov). The values are *d*-band centers integrated from −8 to −4. (g) Transfer of charge from the metal to the support over the oxygen vacancy.

External controls can steer a cluster catalyst across fluxional, coupled or trapped regimes. Two orthogonal levers define this space (Fig. 4a): (i) adsorbate coverage/binding, which sets the chemical residence time *τ*_chem_; and (ii) metal–support coupling, which sets the structural relaxation time *τ*_struct_. Weakening metal–adsorbate interactions (low coverage, inert coadsorbates) shorten *τ*_chem_ and drives the system toward the fluxional regime (*N*_c_ = *τ*_struct_/*τ*_chem_ ∼ 1). Strong binding does the opposite—lengthening *τ*_chem_, effectively locking the adsorbates in place and driving the system toward a coupled or even trapped regime, where the movement of surface atoms is restricted, slowing down the catalytic process. On the structural axis, stronger MSIs (e.g. defect-anchored clusters on reducible oxides) strengthen *τ*_struct_ and bias the system toward coupling, where weak anchoring—or high temperatures that effectively decouple the cluster—shorten *τ*_struct_ and increase fluxionality. In practice, counting coverage and support provides a direct route to *N*_c_ ∼ 1, where reconstruction and turnover are synchronized and metastable isomers persist long enough to function as active sites.

When CO is used as a prototype, higher coverage causes adsorbates to repel each other, which compacts the cluster and shortens the residence time of the adsorbates on the surface (Fig. 4b, Figs S25 and S26). For example, in DFT simulations of Rh_4_, increasing CO loading reduces the fraction of top-layer Rh atoms and increases average Rh–Rh coordination, indicating CO-induced densification (Fig. 4b). The effective CO desorption barrier evolves non-monotonically; it increases from low to moderate coverage and then softens at saturation (from ∼3.11 to ∼1.01 eV). Moreover, the mean reconstruction barrier energy remains at ∼0.50 eV, but the maximum reconstruction barrier energy increases to more than 1.60 eV. Despite the per-molecule softening at monolayer (1 ML), site blocking and electronic pinning by the CO overlayer yield a longer mean residence time (*τ*_chem_↑ at 300 K), whereas *τ*_struct_ remains at ∼10^−3^‒10^−2^ s. We evaluated the CO adsorption capacity on M_5_ (M = Ru, Rh, Pd, Pt) clusters to account for high-coverage effects (*θ* < 1) by grand canonical Monte Carlo (GCMC) simulations. As shown in Fig. S25, the abundance of diverse adsorption sites allows the metal-to-CO stoichiometry to exceed unity. However, lateral CO–CO repulsion and electronic saturation significantly reduce the desorption energy at high loadings. For Pd_5_ and Pt_5_, further adsorption becomes energetically unfavorable above *θ* ∼ 1.6, whereas Ru_5_ has a higher capacity. These results indicate that while high coverage is accessible, the effective coverage is capped at *θ* ∼ 2 for the metals studied, justifying the coverage range explored in our kinetic model. Consequently, the cycle count *N*_c_ = *τ*_struct_/*τ*_chem_ grows from vanishingly small values toward unity, pushing the system into the coupled regime where metastable configurations persist long enough to participate in turnover.

At 300 K, the same coverage effect appears across other metals (Tables S11 and S12). For Ru_5_, Rh_5_ and Pd_5_, increasing CO coverage compacts the clusters and reduces the effective desorption energy, shortening *τ*_chem_ and thereby increasing *N*_c_ from ∼10^−22^ to ∼10^−4^. Pt_5_ shows the same qualitative trend but with a smaller magnitude—coverage modestly perturbs *E*_des_ and keeps *N*_c_ within 10^−7^–10^−5^. These results indicate that coverage-induced densification and the accompanying shift in chemical–structural timescales are broadly general across metals and are not specific to a single element. The increase in *N*_c_ leads to a significant improvement in catalytic activity by 1–2 orders of magnitude, as it brings the system closer to the optimal coupled regime. However, for Pd and Pt, the relatively lower *N*_c_ values prevent them from entering the coupled regime, and thus the improvement in activity is less pronounced (Fig. S26). The enhanced *N*_c_ in metals like Ru and Rh facilitates better synchronization between structural dynamics and reaction events, driving faster CO desorption and improving overall catalytic performance.

We examined how surface crowding and adsorbate–adsorbate interactions tune the coupling between structure and reactivity. On Rh_4_, a full CO monolayer slightly weakens per-molecule binding relative to half-coverage owing to repulsion, yet it effectively locks the cluster: CO–Rh bonds [−2.3 eV, crystal orbital Hamilton population (COHP)] far exceed Rh–Rh metallic bonds (−0.90 eV), so the continuous CO overlayers electronically anchor surface atoms and stabilize metastable geometries (Fig. 4c). Differential charge‒density maps revealed charge accumulation at the Rh–CO interface, confirming strong chemisorption (Fig. 4d). The net result is a shorter CO residence time (faster *τ*_chem_; Fig. S27) and suppressed isomerization (larger *τ*_struct_), which bring the two clocks onto comparable scales (*N*_c_ → 1). Therefore, surface crowding can induce kinetic coupling—often expressed as rate hysteresis and shifts in active-site populations—by pinning fluxional clusters into reactive, non-ground-state morphologies.

To justify the exclusion of hydrogen from our primary kinetic framework, we evaluated the competitive adsorption between CO and H on an Rh_5_ cluster. As illustrated in Figs S28 and S29, CO adsorption consistently outcompetes H due to its superior binding strength. Crucially, as the CO coverage increases from 0 to 1.0 ML, the adsorption energy of hydrogen shifts from −0.72 eV to nearly zero. This destabilization is driven by lateral repulsive interactions and electronic competition within the cluster core. These results confirm that under the CO-rich conditions typical of CO hydrogenation, the cluster surface is dominated by CO, which effectively dictates the structural reconstruction timescales and serves as the representative chemical clock.

Oxide defects can anchor clusters from below much as adsorbates pin them from above. A comparison of Cu*_n_*/TiO_2_(110) with Cu*_n_*/TiO_2−*x*_ (oxygen vacancies) revealed markedly stronger metal–support coupling on the defective surface (Fig. 4e). Vacancies draw more Cu atoms into contact with the metal of the oxide, increasing the MSI, lowering the top layer fraction, flattening the cluster (Fig. S30), reducing network connectivity and increasing *τ*_struct_ from ∼10^−9^ to ∼10^−7^ s; correspondingly, *N*_c_ increases from ∼10^−2^ to ∼1, and the rate increases by two orders of magnitude (Fig. S31 and Table S13). This increase in *N*_c_ is driven by the improvement of metastable configurations, as it brings the system closer to the optimal coupled regime, where the structural dynamics are in alignment with the chemical processes. Electronically, vacancies downshift the Cu *d*-band center (−1.98 → −2.33 eV) and increase interfacial charge transfer (+0.68*e* → +1.09*e*), which is consistent with undercoordinated Ti sites acting as electron acceptors that grip the cluster (Fig. 4f and g). Enhanced coupling between the metal cluster and the support effectively rigidifies the interface, suppressing fluxionality and shifting *N*_c_ from the highly fluxional regime (*N*_c_ ≪ 1) toward the coupled limit (*N*_c_ ∼ 1). This stabilization promotes the formation of more active sites and improves overall catalytic performance.

To evaluate the impact of the support nature on cluster dynamics, we compared the *N*_c_ trends of Cu_5_ supported on reducible TiO_2_(110) and irreducible MgO(111). As summarized in Fig. S32 and Table S14, the weaker MSI on MgO(111) facilitates faster structural reconstruction, reducing *τ*_struct_ and consequently lowering the *N*_c_ value by two orders of magnitude compared with the TiO_2_ system. This shift demonstrates that while the specific *N*_c_ regime depends on support-induced fluxionality, the underlying mechanism of rate enhancement through structural‒chemical synchronization (*N*_c_ ∼ 1) remains universally applicable.

In terms of timescales, coverage and binding primarily set *τ*_chem_, whereas support coupling sets *τ*_struct_ (Fig. 4a). These orthogonal controls define a kinetic phase space. If a system is too fluxional (*N*_c_ ≪ 1), increasing anchoring by adding vacancies or using a stronger-binding support to lengthen *τ*_struct_ and/or increasing coverage or strengthening binding to lengthen *τ*_chem_ will push *N*_c_ toward unity. If a system is trapped (*N*_c_ ≫ 1), weakening anchoring (healing vacancies, selecting a less interactive support) to shorten *τ*_struct_, reducing coverage, increasing binding or decreasing the temperature to lengthen *τ*_chem_, pulls *N*_c_ back toward the coupled regime.

Two fundamental design principles emerge. First, *N*_c_ can be finely tuned by co-adjusting the coverage/binding (which controls *τ*_chem_) and MSIs (which govern *τ*_struct_) to position the catalyst near *N*_c_ ∼ 1 when metastable, reactive motifs are desired, or away from unity when near-thermodynamic ensemble behavior is preferred. Second, the configurational complexity of the catalyst can be optimized by targeting clusters with a moderate number of well-connected isomers—this enables low activation barriers for swift rearrangement, while limited degeneracy helps avoid deep, long-lived traps. These strategies not only optimize the structural dynamics but also significantly enhance catalytic activity by ensuring that the system operates efficiently within the coupled regime, thereby increasing the distribution of highly active sites that drive more effective reactions. Together, these principles—rooted in the concept of structure–reaction dynamic coupling—transform the idea of timescale matching into actionable design strategies. In practice, one can leverage adsorbate crowding (to accelerate the chemistry) and defect-mediated anchoring (to slow structural relaxation) to drive *N*_c_ ∼ 1, stabilize, and exploit metastable structures during turnover. By applying these design principles, catalysts can be engineered to operate within the coupled regime, thereby unlocking a reactivity profile that fully exploits the dynamic landscape of the cluster, rather than relying on a single, static configuration.

## DISCUSSION

Catalysis on supported clusters involves two coupled clocks: the structural relaxation time *τ*_struct_ and the chemical residence/turnover time *τ*_chem_. Their ratio, *N*_c_ = *τ*_struct_/*τ*_chem_, is located when restructuring governs reactivity. The highest leverage occurs near *N*_c_ ∼ 1, where reconstruction and chemistry run on comparable timescales and active metastable isomers persist long enough to enhance activity. This clock-matching perspective rationalizes *operando* hallmarks that static-site models miss—rate hysteresis, non-Arrhenius behavior, broadened/shifted vibrational bands and coverage-dependent selectivity [63,64,67].

The Cu*_n_*/TiO_2_(110) case exemplifies how small size changes reorganize the energy-landscape topology and flip the dynamic regime. Cu_8_ lies deep in the fluxional region (*N*_c_ ≪ 1), rapidly re-equilibrating between reaction events and thus exhibiting near-thermodynamic ensemble reactivity. Adding a single atom to form Cu_9_ shifts the barriers and connectivity enough to yield orders-of-magnitude slower equilibration and *N*_c_ ≫ 1, i.e. kinetic trapping. This ‘Goldilocks’ sensitivity underscores that cluster catalysis is set not only by thermodynamics (isomer energies) but also by the connectivity and heights of the transition network that mediate motion across those states. In practice, such sensitivity helps explain why nominally similar clusters show disparate active sites, spectra and rate laws across labs and conditions; they occupy different regions of the *N*_c_ phase space [33,68].

Metal identity sets the baseline dynamics. Coinage clusters (Cu, Ag and Au) possess narrow isomerization-barrier distributions and weaker CO binding, biasing fast *τ*_struct_ and short *τ*_chem_ to give *N*_c_ ≪ 1 (fluxional, near-thermodynamic ensemble catalysis). In contrast, Rh and Pd feature broader, higher reconstruction barriers and strong CO adsorption, lengthening both clocks but especially *τ*_chem_, which pushes *N*_c_ ≫ 1 and favors long-lived kinetic subensembles. Ru/Pt often sit near the boundary, where modest perturbations (coverage, defects, temperature) tip the system into or out of coupling. These trends connect periodic bonding ‘softness,’ *T*_m_ and adsorption energetics to dynamic state occupation rather than to a single ‘optimal’ static geometry.

Beyond rationalization, the framework is predictive and testable. It anticipates: (i) a defect-density ‘resonance’ with a turnover maximum when metal–support anchoring tunes *τ*_struct_ into resonance with *τ*_chem_; (ii) reversible rate hysteresis and band broadening as *N*_c_ crosses unity under coverage cycling; and (iii) a metal series evolving from fluxional averages (coinage) to trapped states (Rh/Pd), with Ru/Pt maximally tunable. While our mapping uses CO adsorption–desorption as a proxy for *τ*_chem_, additional clocks (diffusion, nucleation, spillover and sintering) can be integrated. The core message stands: design for *N*_c_ ∼ 1 to harness metastable isomers as productive states and engineer adaptive dynamics—tunable *in situ* via coverage, temperature, support chemistry and size.

## CONCLUSION

We establish a predictive framework that orchestrates structure and chemistry for cluster catalysis by coupling two *operando* clocks—the structural relaxation time *τ*_struct_ and the chemical residence time *τ*_chem_—into a single control parameter *N*_c_ = *τ*_struct_/*τ*_chem_. This metric exposes three universal regimes: fluxional (*N*_c_ ≪ 1), kinetically trapped (*N*_c_ ≫ 1) and coupled window (*N*_c_ ∼ 1). It is found that *N*_c_ should be neither too small nor too large. At the fluxional limit (*N*_c_ ≪ 1), structures re-equilibrate too quickly for transient active motifs to persist (negligible gains); in the trapped regime (*N*_c_ ≫ 1), sluggish restructuring strands the catalyst in long-lived, less-active states (hindering turnover), whereas in the coupled window (*N*_c_ ∼ 1), rearrangement is agile yet slow enough for low-barrier sites to survive through turnover and maximize rates. When applied to CO adsorption–desorption on size‐selected Cu*_n_*/TiO_2_(110), the framework yields a master kinetic curve versus *N*_c_, rationalizes pronounced, non-monotonic size effects and unifies *operando* hallmarks—rate hysteresis, non-Arrhenius behavior and broadened/shifted vibrational bands—within a single mechanistic picture.

The analysis provides actionable levers to place catalysts in the desired dynamic state: (i) tune *τ*_chem_ via coverage and adsorbate binding; (ii) tune *τ*_struct_ via metal–support coupling (e.g. defect density); and (iii) select composition/size to set the baseline energy‒landscape connectivity. Trends generalize across metals; coinage clusters prefer fluxionality, Rh/Pd favors trapping and Cu/Pt/Ru often lies near the coupled boundary, where modest perturbations can maximize turnover. In this coupled regime (*N*_c_ ∼ 1), activity peaks as structural flexibility is perfectly aligned with reaction events, allowing for maximum catalytic efficiency and CO desorption rates. Practically, time-scale matching—driving *N*_c_ toward unity for high activity and stability at the same time—emerges as a design rule for stabilizing reactive, non-ground-state configurations during turnover; moving away from unity enforces robustness or suppresses pathway multiplicity when desired. Future work will couple *N*_c_ maps with full microkinetics under realistic feeds and potentials and with data-driven exploration of the composition/support space. By turning *operando* dynamics from a complication into a knob, this study provides a transferable basis for designing adaptive, fluxional catalysts with higher activity and selectivity than static paradigms allow.

## METHODS

All spin-polarized DFT calculations were performed via the GPAW code [69,70]. The projector augmented-wave (PAW) [71] pseudopotentials and the Perdew–Burke–Ernzerhof (PBE) exchange correlation functionals [72] were adopted. The Brillouin zone sampling was restricted to the Monkhorst–Pack [73] 1 × 1 × 1 mesh. An energy cutoff of 400 eV was used in the structure optimization. The geometric structure convergence threshold was set to 10^−4^ eV, with the optimization considered to have converged when the forces on each atom were less than 0.05 eV/Å. To account for the electron localization 3*d* of Ti, the DFT + U method with 4.2 eV [74,75] was employed.

Two O‒Ti‒O layers were employed for determining the structures and potential energy surfaces, with relaxation applied to the topmost layer. A *p*(4 × 2) rutile TiO_2_(110) surface cell was employed. A 15 Å vacuum spacing between adjacent slabs was used to avoid self-interaction.

Transition states were identified with the dynamic NEB method (dy-neb) [76] to produce a good initial guess via the image-dependent pair potential (IDPP) surface method [77] identified with a force tolerance of 0.05 eV/Å. Vibrational mode analysis was also conducted to validate the identified transition states.

More details of the methods, such as the genetic algorithm (GA), KMC, NNPs and MD can be found in the Supplementary data.

## Supplementary Material

nwag072_Supplemental_File

## Data Availability

All calculation methods and data generated and analyzed during the study are provided in the Supplementary data or can be obtained from the corresponding authors upon request. The complete structural and kinetic datasets used in this work are available at https://github.com/chen-jialan/Orchestrating-Structure-and-Chemistry-Dynamics. This record ensures the reproducibility of our automated workflow and provides a validated benchmark for subsequent studies on subnanometer cluster dynamics. The code developed in this work is available at the GitHub page (https://github.com/chen-jialan/Orchestrating-Structure-and-Chemistry-Dynamics).
