# Supplementary material for: Orchestrating structure and chemistry dynamics for cluster catalysis
Source: Natl Sci Rev. 2026 Feb 4;13(7):nwag072. doi: 10.1093/nsr/nwag072 (PMC13070567; doi:10.1093/nsr/nwag072)
Supplement: nwag072_Supplemental_File [file nwag072_supplemental_file.pdf]

Supplementary Information

Orchestrating Structure and Chemistry Dynamics for Cluster Catalysis

Jia-Lan Chen[1,2], Hong-Yue Wang[1,2], Chuan-Liang Ruan[1,2], Jin-Xun Liu*[1,2,3] and Wei-Xue Li*[1,2,3]

[1]State Key Laboratory of Precision and Intelligent Chemistry, University of Science and Technology of China, Hefei, Anhui 230026, China

[2]Department of Chemical Physics, School of Chemistry and Materials Science, University of Science and Technology of China, Hefei, Anhui 230026, China

[3]Hefei National Laboratory, University of Science and Technology of China, Hefei 230088, China

Correspondence and requests for materials should be addressed to J.X.L. (Email: jxliu86@ustc.edu.cn) or to W.X.L. (Email: wxli70@ustc.edu.cn)

Content

1. Supplementary Methodology 3
   1.1 Genetic algorithm (GA) 3
   1.2 Kinetic Monte Carlo 5
   1.3 Neural Network Potential (NNPs) 6
   1.4 Molecule dynamics 8
2. Supplementary Notes 10
   2.1 Supplementary Note 1 10
   2.2 Supplementary Note 2 15
   2.3 Supplementary Note 3 18
   2.4 Supplementary Note 4 19
   2.5 Supplementary Note 5 20
3. Supplementary Figures 22
4. Supplementary Tables 61
5. Supplementary References 76

1. Supplementary Methodology

1.1 Genetic algorithm (GA)

The genetic algorithm (GA) is a classical approach to global optimization.[1-3] The following steps were taken to achieve this objective:

The first step involves the random generation of initial cluster catalyst structures, incorporating reactants and intermediates. Each molecule is assigned a unique tag to distinguish it from other atoms or molecules, with atoms within a molecule bonded and operating as a cohesive unit. The structures are evaluated for the distances between neighboring atoms to prevent strong repulsive forces, which could lead to computational errors. During the GA process, the minimum threshold is set to 25% below the nearest-neighbor distance for the respective material. In general, the optimal population size, denoted as S, should lie within the range of $\log_2(N) < S < 2\log_2(N)$, where N represents the total number of possible combinations in the search space.[4, 5]

Second, the population is updated according to the fitness function, with each structure being assigned a distinct fitness value. The fitness criterion is determined via the following calculation:

$$F_k = \frac{1}{2} \times [1 - \tanh(2\rho_k - 1)] \tag{S1}$$

where ρ_k can be calculated via the fitness function:

$$\rho_k = (E_k - E_{\min}) / (E_{\max} - E_{\min}) \tag{S2}$$

If ρ_k is lower than a randomly generated number between 0 and 1, the structure is discarded. Thirty percent of the lowest-energy structures were retained to maintain the best genes of the parent generation.

Third, the GA relies on crossover and mutation operations, noted by Deaven and Ho,[6, 7] to create new generations. The crossover operation typically involves the exchange of segments of the parent atomic positions or their structural motifs. Moreover, owing to the existence of tags, atoms with the same tag have the same crossover to prevent the disappearance of molecular characteristics.

Mutations are pivotal in the acquisition of efficient 'genes' and the exploration of PESs. In this work, the following mutations were applied specifically to clusters:

1. Rattle mutation: Every atom in the cluster can undergo random movement up to a maximum limit, ensuring that the z positions exceed the support level. Here, the maximum distance is set at 0.6 Å, with 40% of the atoms in the cluster randomly rattled.

2. Rotational mutation:[8] This involves rotating a segment of the cluster around its mass center, ensuring no overlap with the remaining part, which moves an appropriate distance to enrich the configuration diversity, aiding in efficiently escaping local minimum points. In this work, the rotational segment comprises between 1/3 and 2/3 of the atoms on the support.

3. Mirror mutation: Mirror mutation is a specialized mutation technique in which the structure of a cluster is altered by mirroring a part of the cluster along a specific plane or axis.

Structures are discarded by the fitness function on the basis of an interatomic distance comparator[2]. In each cycle, a new structure is generated through crossover and mutation operations, and its interatomic distances are evaluated to prevent the creation of duplicate or unreasonable structures, such as the initial structure assessment. After 100 cycles, if no new optimal structure is found within five consecutive cycles, the structure with the lowest energy is considered the global minimum. Given that GA convergence is dependent on the chosen parameters and initial structures, multiple iterations of GA simulations are performed to ensure the consistency and reliability of the results.

In our GA framework, the initial population size is optimized at 1.5 times the number of atoms (e.g., 6 individuals for Cu_4), with all structures subsequently relaxed via DFT. The evolutionary process uses a crossover probability of 0.9 and a mutation probability of 0.5. Notably, if crossover does not occur, mutation is enforced with a probability of 1.0. The mutation operators are distributed among the mirror, rattle, and rotation moves at a ratio of 1:5:1. To ensure diversity and avoid redundancy, a structural comparator is employed: two configurations are considered identical if their energy difference is within 0.02 eV and their maximum coordinate deviation is less than 0.7 Å. The search converges when the population remains unchanged for three consecutive cycles or after 300 new structures have been generated. These rigorous parameters and stopping criteria ensure a high degree of confidence in the structural coverage of the potential energy surface.

Metastable state structures are selected on the basis of their energy being less than 1 eV relative to the most stable structure. In catalytic systems, particularly those involving clusters, metastable states often govern reaction dynamics, as they can be kinetically accessible and play a significant role in determining reaction rates and selectivity. These states are energetically close to the global minimum, meaning that they can be easily accessed during the catalytic process without requiring excessively high activation energies. By focusing on metastable states that are within this energy range, we capture the essence of realistic catalytic conditions, where the system may be kinetically trapped in local minima rather than being at thermodynamic equilibrium. This selection ensures that the chosen structures represent feasible configurations in the context of dynamic, complex reaction networks, providing a more accurate description of the catalytic behavior and pathways.[9, 10]

1.2 Kinetic Monte Carlo

In this work, for the simulation of elementary reaction times, a kinetic Monte Carlo (KMC) simulation is employed.[11]

In a kMC simulation, we are interested in all possible elementary events that may bring state ω to a new possible state. The probability that the system has escaped from state ω at some time less than or equal to τ is given by

$$p_{escaped} = 1 - \exp(-k_{tot}\tau) \tag{S3}$$

where k_{tot} is the total escape rate from state ω due to all possible elementary events, which is calculated below:

$$k_{tot} = \sum_{w \neq w'} k_{ww'} \tag{S4}$$

where $k_{ww'}$ is the escape rate from state ω to ω'. The probability density function of a transition occurring at time $t + \tau$, given that the system is at state ω at time t, is given by the following distribution:

$$p(\tau) = k_{tot} \exp(-k_{tot}\tau) \tag{S5}$$

In addition, $p_{ww'}$ is calculated as follows:

$$p_{ww'}(\tau) = k_{ww'} \exp(-k_{ww'}\tau) \tag{S6}$$

After invoking the inversion generation method,[12] the escape times are calculated as follows:

$$t_{ww'} = -\ln(1-u)/k_{ww'} \tag{S7}$$

where u is a random number from 0 to 1. Essentially, this equation calculates the time it takes for the system to move from one state to another on the basis of the escape rate and a randomly generated value, providing a key component in modeling such dynamic systems.

In this work, the cluster states are selected from a comprehensive database of cluster isomers, ensuring a systematic exploration of the configurational space. The selection process relies on the similarity of clusters, quantified via the root mean square deviation (RMSD), with a threshold of 4.0 Å, which ensures that only isomers with structurally similar characteristics are considered. To validate the 4.0 Å RMSD threshold, we performed sensitivity analyses on representative clusters (Cu_5 and Cu_9) using expanded cutoffs of 4.5 Å and 5.0 Å. As summarized in **Supplementary Table 15**, increasing the threshold marginally expands the reaction network but yields no qualitative shift in kinetic timescales or N_C trends. Specifically, Cu_5 maintains its high-activity profile ($G > 10$), and Cu_9 remains in the trapped regime ($G < 1$). These results demonstrate that the derived structure–kinetics relationships are independent of the specific RMSD cutoff choice. Therefore, this criterion facilitates the identification of closely related states that are likely to be interconnected through potential transitions. Additionally, the selection is further refined by calculating the reconstruction barrier energy ($E_{a,recon}$), which provides insight into the energy required for transitioning between different isomeric states. To assess the dynamic feasibility of these transitions, the escape rate ($k_{ww'}$) is also calculated, reflecting the likelihood of overcoming the reconstruction barriers within a given timescale. Together, these factors—structural similarity, barrier energy, and escape rate—serve as a robust framework for identifying relevant metastable states, ensuring that only energetically and kinetically feasible isomers are selected for further analysis, ultimately providing a more realistic representation of the system's catalytic behavior.

1.3 Neural Network Potential (NNP)

In this study, the neural network potential (NNP) is constructed based on an SO(3)-equivariant graph neural network (GNN). The architecture is designed such that the output layer yields a scalar

value representing either the atomic energy or the atomic work function. The complete NNP workflow is executed through four primary stages:

1. **Initialization of Node and Edge Features**: Atomic node features $h_v^0(q)$ are integrating local atomic descriptors, while edge features $S_m^l(\vec{r}_{ij})$ —representing pairwise relative positions—are encoded via equivariant convolutional filters to capture the initial geometric environment.

2. **Local Message Passing:** At each iteration t, a messages m_t is computed for each atom by aggregating information from its local neighborhood:

$$m_v^t(q) = \sum_{\mu \in \mathrm{N}(\mu)} M_t(h_v^{t-1}(q), S_m^l(\vec{r}_{ij})) \tag{S8}$$

$$S_m^l(\vec{r}_{ij}) = R(\vec{r}_{ij}) Y_m^l(\vec{r}_{ij}) \tag{S9}$$

$$R(r_{ij})\Big|_{i=1}^{N} = 1/Z \times f(r_{ij})\Big|_{i=1}^{N} \tag{S10}$$

M_t is a learnable message function involving a geometric tensor product ⊗ between atomic features and convolution filters:

$$M_t(h_v^{t-1}, S_m^l(\vec{r}_{ij})) = \frac{1}{\sqrt{z}} h_v^{t-1} \otimes S_m^l(\vec{r}_{ij}) \tag{S11}$$

These products are evaluated using Clebsch–Gordan coefficients[13] to ensure SO(3) symmetry, maintaining the rotationally equivariant nature of the feature representations.

3. **Node feature update**: Following message aggregation, atomic features are updated as follows:

$$h_v^t = U_t(h_v^{t-1}(q), m_v^t(q)) \tag{S12}$$

The update function U_t is implemented as a multilayer perceptron (MLP) that integrates the preceding atomic state with the incoming message. These updated features effectively encode both structural geometry and potential-dependent electronic effects. Notably, our model maintains static edge features throughout the iterations, prioritizing node-level feature refinement.

4. Global Graph Readout and Physical Prediction: After several rounds of message passing, a global representation of the graph is constructed via symmetry-aware pooling. The total potential energy E is predicted by:

$$E(\mathbf{r}) = \oplus_{\Omega} A(h_v^t) \tag{S13}$$

where $\oplus$ represents the global pool, where the sum function is applied to compute the energy. A is also the multilayer perceptron. In the implementation of GNN, the output layer has only one representing the potential energy. The MPNN is expressed as follows:

$$\begin{aligned} E(\mathbf{r}) &= \oplus A(h_v^t) = \oplus A((U_t(h_v^{t-1}, m_v^t))) \\ &= \oplus A((U_t \circ U_{t-1} \circ \ldots \circ U_1(h_v^0, m_v^0))) \\ &= \oplus A((U_t \circ U_{t-1} \circ \ldots \circ U_1(\sum_{\mu \in \mathrm{N}(\mu)} m_1(h_v^0(c), S_m^l(\vec{r}_{ij}))))) \end{aligned} \tag{S14}$$

Therefore, the NNPs architecture is realized.

The neural network potential (NNP) for the Cu/TiO_2 system was trained on a comprehensive dataset of 106,700 structures generated via ab initio molecular dynamics (AIMD) and genetic algorithm (GA) searches. The architecture utilized a cutoff radius of 4.5 Å, with $l_{\max}$ and 3 iterations, incorporating two hidden layers of 64 nodes each. The optimized NNP achieved a high degree of fidelity, characterized by a root-mean-square error (RMSE) in energy of 2.97 meV/atom.

1.4 Molecule dynamics

In molecular dynamics simulations, it is often useful to reduce large number of degrees of freedom of a physical system to study the part we considered. In this work, the system of study is focused on the cluster fluxionality and molecule reaction. Therefore, enhancing sampling in molecular dynamics simulations are employed and reconstruct the free-energy surface (FES) as a function of few selected degrees of freedom, referred to as collective variables (CVs).

In this work, collective variables module (Colvars)[14] of LAMMPS[15] is employed as the implementation of sampling algorithms. The CV of adsorption/desorption is set as the distance of the center of molecule and cluster. Moreover, extended-system adaptive biasing force (eABF)[16-18] and metadynamics (MTD)[19] are employed as a biasing method. The Nose–Hoover thermostat is

employed for NVT ensemble sampling at 500 K to achieve the efficient calculation of potential of mean force (PMF).

2. Supplementary Notes

2.1 Supplementary Note 1

The coupling of kinetic processes in adsorption and desorption at equilibrium is central to understanding catalytic reactions. The rate of catalytic reactions can be expressed by the following general rate equation:

$$r = \sum_i (p_i \times r_i) \tag{S15}$$

where r_i denotes the individual reaction rate for each state, and p_i is the probability of the system occupying state i, which is determined by the energy distribution of adsorbates. Under thermodynamic equilibrium, the probability p_i follows the Boltzmann distribution:

$$p_i = \exp\left(-\frac{E_{R,i} - E_{\mathrm{stable}}}{k_b T}\right) \tag{S16}$$

where $E_{\mathrm{R},i}$ is the energy of the adsorbed molecules in state i, E_{stable} is the stable state energy, k_b is the Boltzmann constant, and T is the temperature.

Moreover, the rate r_i of a specific isomer is determined by the balance of the desorption and adsorption processes. Specifically, the desorption rate constant k_{des} dictates the rate at which adsorbed molecules (R) depart from the surface, whereas the adsorption rate constant k_{ads} controls the rate at which molecules attach to the surface on the basis of the adsorption–desorption equilibrium ([R]€ R+*)

$$r_i = r_+ - r_- = k_{\mathrm{des}}[\mathrm{R}] - k_{\mathrm{ads}} \frac{p_R}{P^\theta} \tag{S17}$$

where r_+ and r_- are the forward and reverse reaction rates, respectively., respectively. On the basis of collision theory, the adsorption and desorption rate constants are given by:[20, 21]

$$k_{\mathrm{ads}} = \frac{PA}{\sqrt{2\pi m k_b T}} S \tag{S18}$$

$$k_{\mathrm{des}} = \frac{k_b T^3}{h^3} \cdot \frac{A(2\pi m k_b)}{\sigma\theta_{\mathrm{rot}}} \exp\left(-\frac{\Delta E_{\mathrm{des}}}{k_b T}\right) = \lambda \times T^3 \exp\left(-\frac{\Delta E_{\mathrm{des}}}{k_b T}\right) \tag{S19}$$

where λ is a preexponential factor and ΔE_{des} is the desorption activation energy. P, m, A, and S represent the partial pressure and mass of the adsorbate in the gas phase, the surface area of the adsorption site, and the sticking coefficient, respectively. In this work, S and A are set to 1 and 10^{-20} m^2, respectively. These constants describe the rates of adsorption and desorption, which are dependent on both temperature and pressure.

Moreover, on the basis of transition state theory, the reaction and reconstruction rates are calculated by:

$$k_a = A_a e^{-\frac{E_a}{k_b T}} \tag{S20}$$

where E_{a} and A_{a} are the barrier energy and prefactor of the surface reaction and reconstruction, respectively. In this work, A_{a} is 10^{13}.

At equilibrium, the rates of adsorption and desorption are equal, ensuring that the system maintains a steady-state surface coverage. This condition of equilibrium allows for the simplification of the rate expression when P_{R} is small:

$$r = \lambda T^3[\mathrm{R}] \times \sum_i \exp\left(-\frac{E_{ads,i} - E_{\mathrm{stable}} + \Delta E_{\mathrm{des},i}}{k_B T}\right) \tag{S21}$$

However, in real catalytic processes, the system often operates out of equilibrium, leading to the coupling of adsorption and desorption rates, which are influenced by dynamic surface reorganization, such as cluster formation. Surface clusters, which are composed of atoms or molecules that dynamically reorganize during catalytic reactions, can modify the adsorption/desorption energy landscape, thereby altering the rate constants. In nonequilibrium scenarios, the coupling between adsorption and desorption can be expressed as:

$$r = \lambda T^3[\mathrm{R}] \times \sum_i p_i \exp\left(-\frac{\Delta E_{\mathrm{des},i}}{k_B T}\right) \tag{S22}$$

where p_{i} denotes the probability of a particular nonequilibrium state, and the system's surface coverage, denoted as **(R)**, is controlled by the balance between adsorption and desorption. The coupled kinetics reflect the feedback mechanism between the adsorption rate, desorption rate, and dynamic reorganization of the catalyst surface, ultimately influencing the overall reaction rate.

To refine this expression, we incorporate the temporal dynamics associated with each state:

$$\begin{aligned} r(t) &= \sum_i (p_i \times r_i) = \sum_i p_i(\tau_{esp,i}) \times r_i \\ &= \lambda T^3 [\mathrm{R}] \times \sum_i p_i(\tau_{esp,i}) \exp\left(-\frac{\Delta E_{\mathrm{des},i}}{k_B T}\right) \end{aligned} \tag{S23}$$

In this revised expression, the probability $p_i(t)$ is replaced by the residence time of the rate-limiting step over structure dynamics, denoted as τ_{esp}, which incorporates the temporal aspect of the adsorption–desorption equilibrium. The residence time τ_{esp} is defined as the minimum between the reconstruction time and the reaction time:

$$\tau_{\mathrm{esp}} = \min(\tau_{\mathrm{struct}}, \tau_{\mathrm{chem}}) \tag{S24}$$

where τ_{struct} and τ_{chem} are the times for cluster reconstruction and for the reaction to occur, respectively. The residence time is further described as:

$$\tau = -\frac{\ln(1-u)}{k} \tag{S25}$$

where u is a random number between 0 and 1 and where k is the rate constant. Therefore, these two times are calculated as follows:

$$\tau_{\mathrm{struct}} = -\frac{\ln(1-u)}{k_{\mathrm{struct}}} \tag{S26}$$

$$\tau_{\mathrm{chem}} = -\frac{\ln(1-u)}{k_{\mathrm{chem}}} \tag{S27}$$

where k_{struct} and k_{chem} are the rates for cluster reconstruction and chemical reactions, respectively, according to **Supplementary Formulas S11–S13**. To establish the true thermodynamic timescale, we can derive the relationship for the intermediate timescale as follows:

$$\tau_{\mathrm{equ}} = \sum_{n=1}^{N} 1/r(t,n) \tag{S28}$$

The total equilibrium time, τ_{equ}, is determined by summing the inverse of the rate at each intermediate state across the entire system. To determine the number of steps required to achieve equilibrium, we use the following equation:

$$\min\left\{N \,\middle|\, \sum_{n=1}^{N}\tau_{\text{esp}}(n) \ge \tau_{\text{equ}}\right\} \tag{S29}$$

This equation aims to identify the minimum number of steps, *N*, for which the cumulative residence time exceeds or equals the equilibrium time.

Throughout each catalytic cycle, the reaction rate typically exceeds the surface reconstruction rate, causing the equilibrium configuration to evolve dynamically. As the reaction progresses, the speed of reconstruction slows down, which is reflected in the decreasing rate $r(t, n)$. This deceleration in the structural equilibration process suggests that the system becomes progressively sluggish over time. However, the precise timescale for this slowdown remains uncertain. The term t_{equ} represents the reconstruction time for each catalytic cycle, offering insight into the dynamic nature of the catalyst during the reaction.

On the basis of the preceding expressions, we can estimate how dynamic coupling can increase the activity of a Cu cluster. From the equation, we observe that the timescale to achieve equilibrium when t_{equ}(n) is the constant, denoted as *N*, is given by:

$$N = \max\left(\frac{\tau_{\text{struct}}}{\tau_{\text{chem}}},1\right) = \max\left(\frac{\tau_{\text{struct}}}{\tau_{\text{des}}},1\right) \tag{S30}$$

where τ_{chem} is the characteristic time related to the adsorption–desorption equilibrium in this work, which can be expressed as:

$$\tau_{chem} = -\frac{\ln(1-u)}{\lambda \times T^3 \exp\left(-\frac{\Delta E_{\text{des}}}{k_b T}\right)} \tag{S31}$$

Substituting the expression for τ into the equation for *N*, we obtain:

$$N = \max\left(\frac{\tau_{\text{struct,equ}} \times \lambda T^3 \exp\left(-\frac{\Delta E_{\text{des}}}{k_b T}\right)}{-\ln(1-u)},1\right) \tag{S32}$$

where $\tau_{struct,equ}$ is the cluster equilibrium time, which is calculated via **Supplementary Formula S19**. When considering a continuous process in an unlimited system, N modulates the probability. Thus, we define the external lever N_c, expressed as:

$$N_c = \tau_{\text{struct}} / \tau_{\text{chem}} = (\sum_{i=1}^{m} N) / m \quad \text{(S33)}$$

where m is the number of isomers.

This expression (**Supplementary Formulas S25–26**) provides valuable insight into the timescale of the catalytic process, accounting for both the equilibrium time and the dynamic coupling between the adsorption and desorption processes. Importantly, when τ_{equ} is constant, an increase in temperature influences the term $T^3 \times exp(-\frac{\Delta E_{\text{des}}}{k_b T})$, which is involved, as both components are temperature dependent.

To accurately map the potential energy landscape of CO-adsorbed clusters, we employed a global optimization framework with full structural relaxation. Unlike sampling methods that fix the metal core, our approach relaxes all atomic coordinates—including both the CO molecules and the metal atoms—simultaneously. This is critical because the coordination of CO induces significant cluster reconstruction, yielding geometries that differ fundamentally from their bare-cluster counterparts. By avoiding frozen constraints, we ensure that the reported kinetic networks and N_C values are based on the most thermodynamically relevant configurations under reaction conditions.

As the temperature increases, both the factor T^3 and the exponential term $\tau_{\text{equ}} \times \lambda T^3 \exp\left(-\frac{\Delta E_{\text{des}}}{k_b T}\right)$ increase. This results in a stronger coupling between the adsorption and desorption processes. The increased temperature enhances the kinetic energy of the molecules, leading to faster adsorption and desorption rates. This intensified dynamic coupling accelerates the catalytic cycle, thereby improving the overall catalytic activity of the cluster.

In essence, temperature not only increases the overall reaction rate but also enhances the coupling between the adsorption–desorption equilibrium and the dynamic reorganization of the

cluster surface. This synergy results in a more efficient catalytic process, where the system reaches equilibrium more rapidly, leading to enhanced catalytic performance.

In conclusion, the coupling of adsorption–desorption processes with the dynamic reorganization of the catalyst surface plays a pivotal role in determining the reaction rate and the timescales associated with system equilibration. By integrating these temporal dynamics into the kinetic model, we can gain a more comprehensive and accurate understanding of the catalytic process under nonequilibrium conditions.

2.2 Supplementary Note 2

In this study, we present a method for calculating the ratio N_c, which quantifies the relationship between the structural and chemical reaction escape times in cluster catalytic systems. The calculation follows a multistep approach, as outlined in the flowchart in **Supplementary Figure 1**. The process includes the use of genetic algorithms (GAs), reaction pair recognition, simulations, and kinetic Monte Carlo (KMC) simulations to derive the structural and chemical reaction escape times (τ_{struct} and τ_{chem}), which are then used to compute N_c.

1. **Initial Population Generation**

   The process begins with the generation of the **initial population**. This step involves the creation of a dataset comprising various candidate structures or configurations. These candidates represent potential initial states for the subsequent steps of optimization and analysis.

2. **Cluster Dataset Creation and Metastable Dataset**

   Next, the **Cluster Dataset** is generated from the Initial Population via the GA. This dataset consists of a structure derived from the clusters, which are essential for exploring the system's behavior. Additionally, we apply an energy window cutoff condition of $E_{window} < 1$ eV to obtain a metastable dataset, which excludes structures that fall outside this energy range to ensure that only physically relevant configurations are considered. Moreover, we employ a dual-criterion approach to identify symmetrically equivalent structures. First,

energy degeneracy is assessed within a 0.02 eV window. Second, structural similarity is quantified via the embedded atom descriptor[22] combined with the REMatch kernel[23]:

$$K(A,B) = \mathbf{Tr}\mathbf{P}^{\alpha} C(A,B)$$
$$\mathbf{P}^{\alpha} = \underset{P \in U(N,N)}{\arg\max} \sum_{ij} P_{ij}(1 - C_{ij} + \alpha P_{ij}) \tag{S34}$$

where the coupling matrix **P** minimizes the regularized overlap energy between local environments C_{ij} in structures A and B. This kernel is strictly invariant to translation, rotation, and permutation. Configurations exhibiting a similarity distance $K < 0.01$ are classified as identical isomers, ensuring the kinetic network's integrity.

3. Structure Pair Generation and Reconstruction Barrier Dataset

A critical step involves the generation of **Structure Pairs** and the **Reconstruction Barrier Dataset for networks**. In this step, **the root mean square deviation (RMSD)** is computed to assess the similarity between the cluster configurations. To address atom-indexing ambiguity, we employ a permutationally best-matched RMSD approach. This method exhaustively evaluates all possible atom permutations to identify the minimum geometric distance between configuration pairs, ensuring that the metric is physically rigorous. The RMSD serves as a measure of the structural similarity of the clusters, ensuring that only configurations with less than 4 Å deviation from the reference structures are retained for further analysis. This method exhaustively evaluates all possible atom permutations to identify the minimum geometric distance between configuration pairs, ensuring the metric is physically rigorous. Then, **the transition states** are calculated **via structure pairs, and the reconstruction barrier dataset is obtained**. These states are pivotal in understanding the energy barriers that need to be overcome during structural transitions in the system.

4. Kinetic Monte Carlo (KMC) simulations

From the structure pairs and transition state data, we construct the **reconstruction barrier dataset**. This dataset includes energy barriers associated with the reconstruction of the catalyst's surface structure. To simulate the transition kinetics and determine the structural escape time (τ_{struct}), we employ **kinetic Monte Carlo (KMC)** simulations. KMC provides a

probabilistic framework to model the time evolution of these transitions, providing insights into the system's dynamic behavior and leading to the computation of τ_{struct}.

5. **Reaction dataset and chemical temperature calculation**

   The reaction dataset is created by considering the adsorption and desorption processes, which are key reactions in the catalytic system. We again apply **KMC simulations** to model the dynamics of these reactions, resulting in the calculation of the chemical reaction escape time (τ_{chem}), which represents the equilibrium associated with the chemical reactions.

6. **Calculation of N_c**

   Finally, we compute the ratio N_c. This ratio provides a measure of the balance between structural and chemical dynamics within the catalytic system.

In summary, our study employs a standardized four-stage protocol to construct cluster isomerization networks: (i) PES sampling via a genetic algorithm; (ii) isomer pairing using a permutationally optimized RMSD criterion; (iii) automated TS location with rigorous pathway verification; and (iv) evaluation of τ_{struct} and N_c through microkinetic modeling and KMC simulations. This pipeline ensures that the structural–chemical synchronization is evaluated with high fidelity across diverse metallic systems. Moreover, We utilized the NetworkX package[24] to architect the isomerization networks from the identified isomer connectivity. To bridge the gap between static energetics and dynamic behavior, forward and backward rate constants derived from transition state theory were integrated into a microkinetic model. The structural equilibration time (τ_{struct}) was then extracted under random initial distribution conditions. This automated pipeline ensures a rigorous, reproducible translation of potential energy surface (PES) data into macroscopic kinetic timescales.

To provide an intuitive understanding of the structural fluxionality, we have included representative network visualizations for all studied metal clusters (**Supplementary Figures 34–40**). These diagrams map the isomerization architecture for Cu, Ag, Au, Ru, Rh, Pd, and Pt, explicitly showing the connectivity between isomers through identified transition states. These visualizations illustrate the topological complexity of the potential energy surface and clarify the pathways governing the structural equilibration timescales reported in our kinetic analysis.

2.3 Supplementary Note 3

The temperature dependence of equilibrium establishment can be derived through fundamental thermodynamic and kinetic principles. The van't Hoff equation describes the equilibrium constant's temperature dependence:

$$\frac{d\ln K_{\text{equ}}}{dT}=\frac{\Delta H^{\circ}}{RT^{2}} \tag{S35}$$

Its integrated form reveals the thermodynamic parameters:

$$\ln K_{\text{equ}}=-\frac{\Delta G^{\circ}}{RT}=-\frac{\Delta H^{\circ}}{RT}+\frac{\Delta S^{\circ}}{R} \tag{S36}$$

For kinetic considerations, the Arrhenius equation governs the rate constant:

$$k=Ae^{-\frac{E_a}{RT}} \tag{S37}$$

The equilibrium establishment time (t_{equ}) is inversely proportional to the rate constant. This relationship yields:

$$\tau_{\text{equ}}\propto\frac{1}{k}\Rightarrow\tau_{\text{equ}}=C\cdot e^{\frac{E_a}{RT}} \tag{S38}$$

Linearizing this expression provides a practical form for experimental validation:

$$\ln\tau_{\text{equ}}=\ln C+\frac{E_a}{RT} \tag{S39}$$

The temperature sensitivity of the equilibrium time between two temperatures is as follows:

$$\frac{t_{\text{equ}}(T_1)}{t_{\text{equ}}(T_2)}=e^{\frac{E_a}{R}\left(\frac{1}{T_1}-\frac{1}{T_2}\right)} \tag{S40}$$

Structural Complexity Considerations

We propose that configurational entropy may induce nonmonotonic behavior in a temperature-dependent manner. Three distinct regimes emerge:

1. Moderate entropy regime: Dynamic coupling dominates

2. Low-entropy regime: Reaction kinetics control

3. High-entropy regime: Structural dynamics prevail

This theoretical framework was applied to investigate the dynamic behavior of metal clusters (n=5–10) supported on TiO_2(110) surfaces. The derived empirical relationship demonstrates excellent agreement with the computational results:

$$\log(\tau_{\text{equ}}) = a \times 1000 / T + b = k / T + b \tag{S41}$$

where τ_{equ} represents the stabilization time and T represents the absolute temperature. *a* and *b* are system-specific coefficients obtained from linear regression analysis.

2.4 Supplementary Note 4

To quantify the structural complexity of clusters during reconstruction, we introduce a configurational entropy metric (*H*) defined as:

$$H = N / A_i^2 = N / [i \times (i-1)] \tag{S42}$$

where *N* represents the network connectivity and *i* is the number of isomers. This descriptor correlates linearly with the intercept (*b*) of the equilibrium time equation.

The temperature-dependent relaxation dynamics further depend on the isomer ensemble complexity. We propose that the rate constant (*k*) follows a volcano-type relationship with the number of metastable states (*N*), described by:

$$k = a_2 \times |N - \alpha| + b_2 \tag{S43}$$

$$b = a_1 \times H + b_1 \tag{S44}$$

where a_2 = 0.097, b_2 = 0.10, and the optimal α = 58. This nonmonotonic trend suggests that reconstruction kinetics are maximized for systems with intermediate metastability.

Combining these effects, the effective energy barrier for reconstruction is derived as:

$$\log(\tau_{\text{equ}}) = \frac{1000}{T}\left(a_2 |N - \alpha| + b_2\right) + a_1 H + b_1 \tag{S45}$$

For a fixed temperature, this simplifies to:

$$\log(\tau_{\text{equ},T}) = \underbrace{\left(\frac{a_2 \cdot 1000}{T}\right)|N - \alpha|}_{\text{Metastability term}} + \underbrace{\frac{a_1 N}{i(i-1)}}_{\text{Complexity term}} + \underbrace{\left(\frac{b_2 \cdot 1000}{T} + b_1\right)}_{\text{Baseline}} \tag{S46}$$

Key Implications

1. **Role of *N***: For N <58, increasing N reduces $|N - \alpha|$, shortening τ_{equ} owing to the negative prefactor a_2.
2. **Role of *i***: larger isomer counts (i) increase H, but the positive a_1 term dominates, prolonging τ_{equ}.
3. **Temperature dependence**: The metastability term scales inversely with T, whereas the complexity term remains T-independent.

This model establishes a quantitative link between cluster topology (N, i), configurational entropy (H), and reconstruction kinetics, offering design principles for dynamic catalysts.

2.5 Supplementary Note 5

The structural relaxation time (τ_{struct}) decreases exponentially with increasing reduced temperature (T/T_m), exhibiting a strong inverse correlation (R^2 = 0.83), which is expressed below:

$$\tau_{struct} = a \times T / T_m + b \tag{S47}$$

where the slope (a) and intercept (b) are −12.08 and −2.05, respectively.

The fitted slope indicates that the structural dynamics of metal clusters are highly sensitive to thermal excitation normalized by their melting temperature. Metals with lower T/T_m ratios, such as Ru, retain sluggish atomic rearrangements, which is consistent with strong metallic bonding and limited configurational entropy. In contrast, Ag, positioned near the high T/T_m regime, exhibits fast structural fluctuations indicative of enhanced atomic mobility.

This temperature scaling behavior implies that the timescale of structural dynamics is fundamentally governed by the reduced thermal energy relative to the cohesive strength of the metal. This correlation provides a quantitative framework linking the thermodynamic stability of clusters to their fluxionality. The observed high degree of atomic mobility well below the T_m is particularly crucial for understanding the behavior of coinage metals in catalytic environments, where facile atomic rearrangement dynamically reshapes the active sites and directly modulates the reaction kinetics.

3. Supplementary Figures

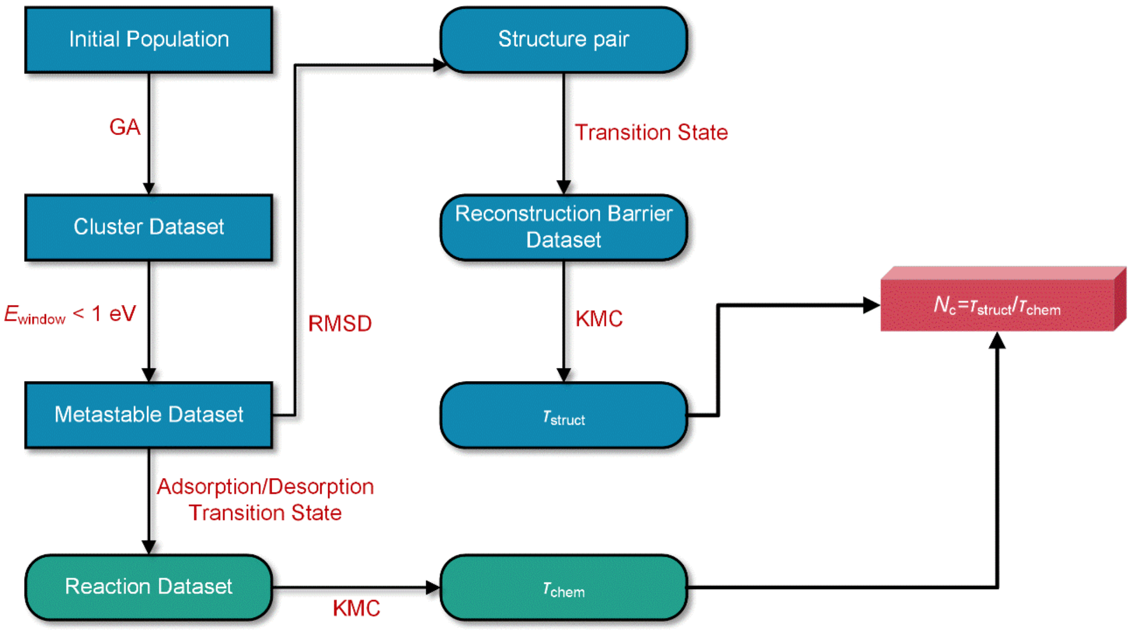

Supplementary Figure 1. Flowchart of the N_c calculation methodology. This diagram illustrates the step-by-step procedure for computing the ratio N_c, which quantifies the relationship between the structural and chemical escape times in catalytic systems. The process begins with the generation of the initial population, the creation of a metastable dataset, and the reconstruction and chemical reaction dataset for networks. Kinetic Monte Carlo (KMC) simulations are applied to calculate the structural escape time τ_{struct} and chemical escape time τ_{chem}, which are then used to compute the final ratio.

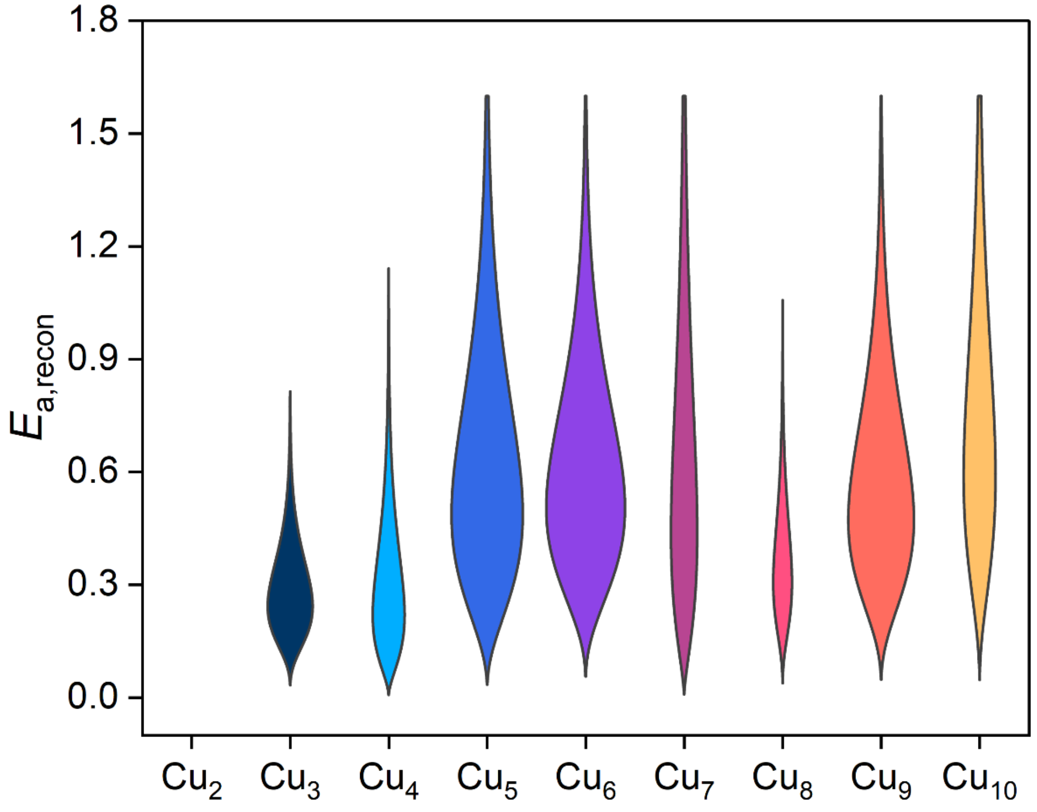

Supplementary Figure 2. Distribution of forward reconstruction barriers ($E_{a,recon}$) for the Cu_2–Cu_{10} clusters. Violin plots illustrate the statistical spread of forward transition-state energies associated with structural reconstruction events. The results reveal cluster size-dependent variations in both the median and distribution width of $E_{a,\ recon}$, reflecting structural fluxionality and the configurational complexity of the potential energy landscape. The unit is eV. This notation is used throughout the Supplementary Information.

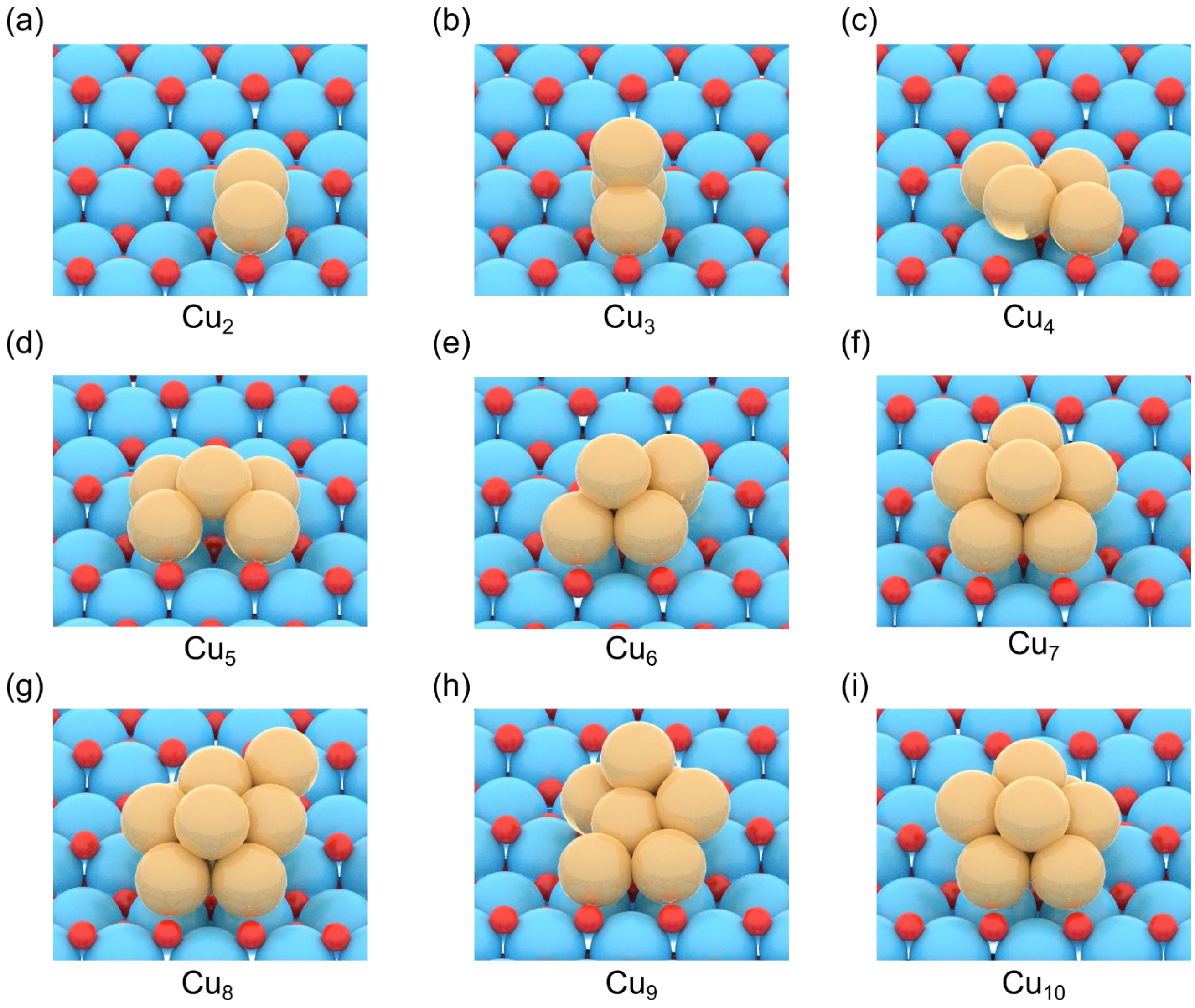

Supplementary Figure 3. Representative Cu_n/TiO_2 structures with increasing clusters (n = 2–10). Configurations with varying numbers of Cu clusters supported on TiO_2(110). The blue and red spheres represent Ti and O atoms, respectively. This notation is used throughout the Supplementary Information. Yellow spheres denote Cu atoms.

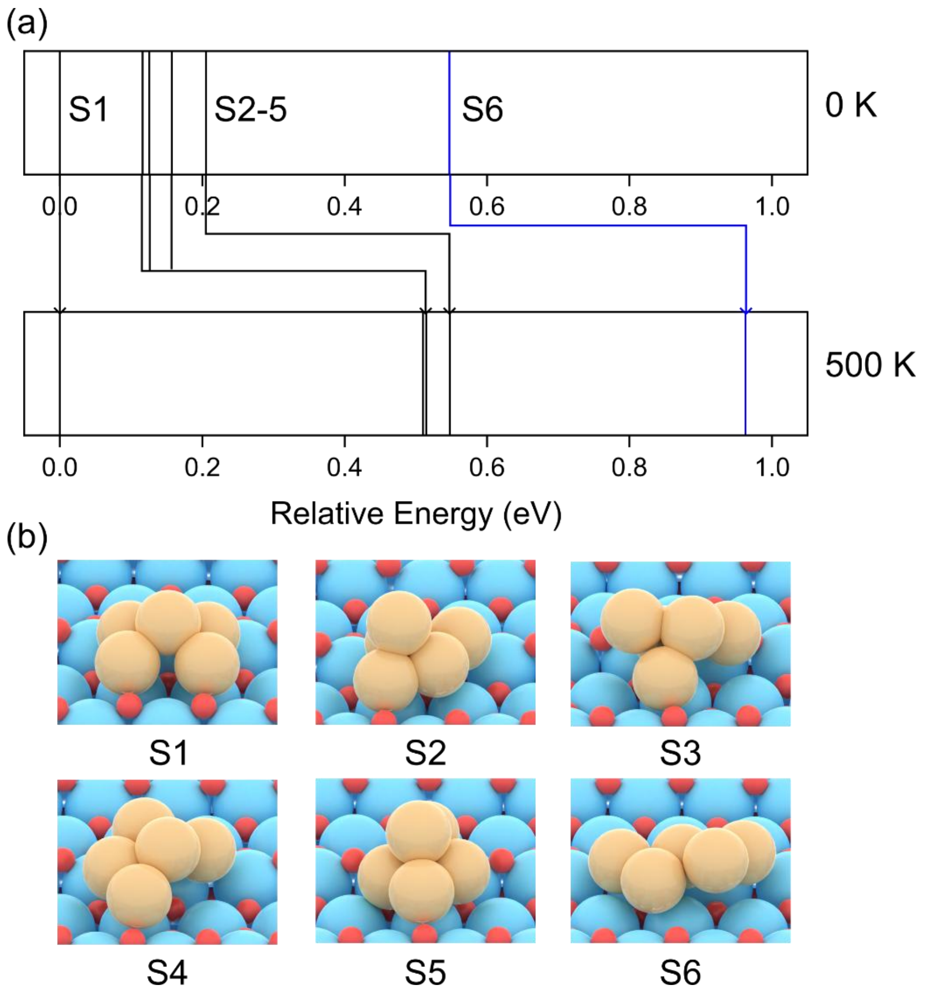

Supplementary Figure 4. Schematic free-energy pathway and representative configurations of the Cu_5/TiO_2 clusters at different temperatures. (a) Schematic illustration of the free-energy profile along the reaction coordinate, highlighting sequential stabilization and transformation steps between metastable states at different temperatures. (b) Representative atomic configurations of the Cu_5 cluster supported on the TiO_2 surface corresponding to distinct minima along the path, illustrating structural rearrangements of the cluster and its interaction with the oxide support.

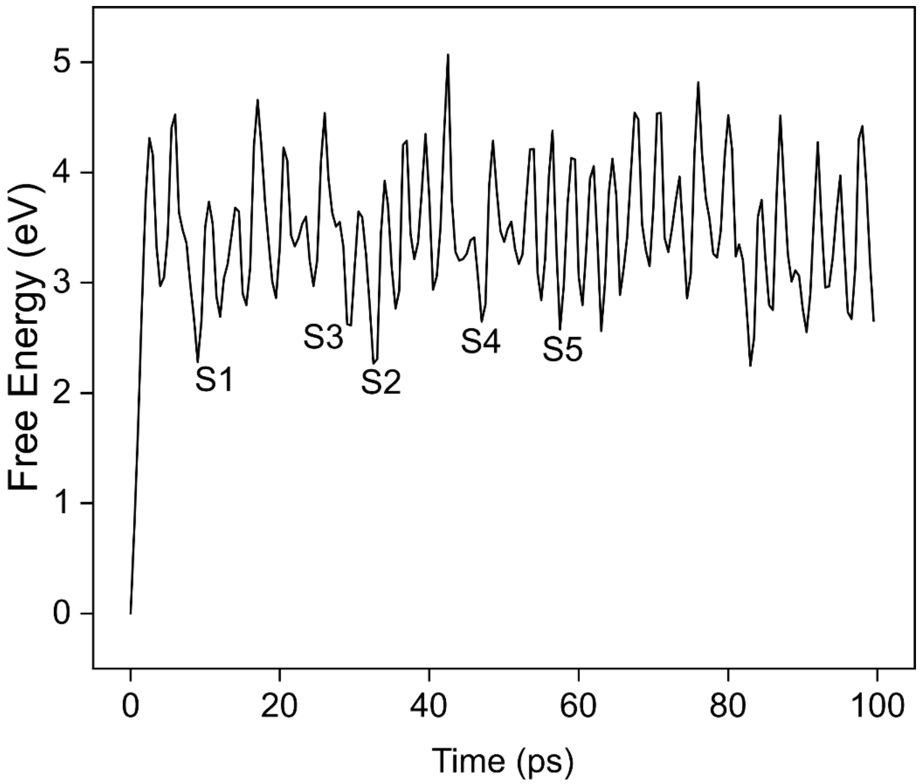

Supplementary Figure 5. Free-energy evolution along the simulation trajectory and identification of representative states. Free energy (eV) as a function of time (ps) obtained from the sampled trajectory over 0–100 ps at 500 K. After an initial relaxation, the system explores a rugged free-energy landscape with pronounced fluctuations. Labels S1–S5 mark representative metastable basins (local minima) selected from the trajectory to illustrate distinct structural states accessed during sampling.

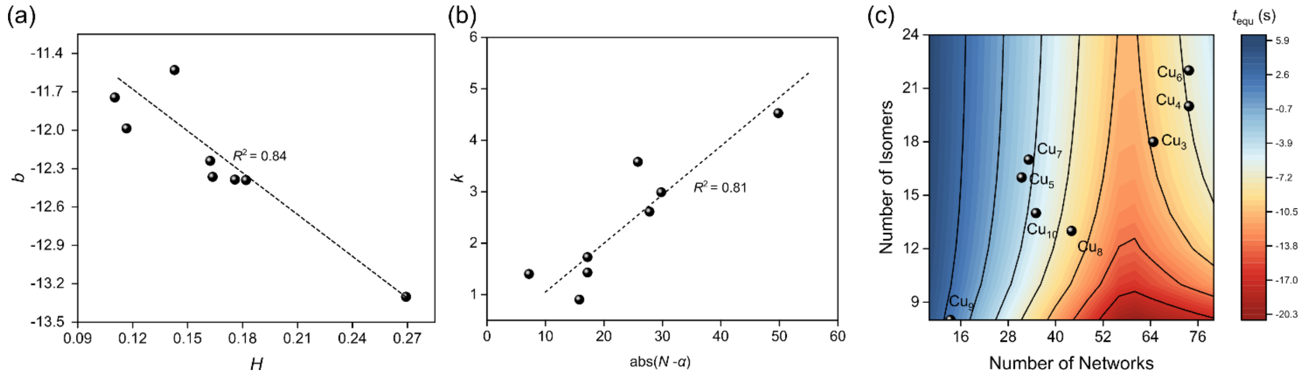

Supplementary Figure 6. Relationship between the configuration and reconstruction equation time (**a**) Relationship between the intercept and network connection descriptors (*H*). (**b**) Relationships between slope and descriptors over the number of isomers (*N*). (**c**) 2D phase diagram of the thermodynamic equilibrium time with respect to the number of isomers and the number of networks.

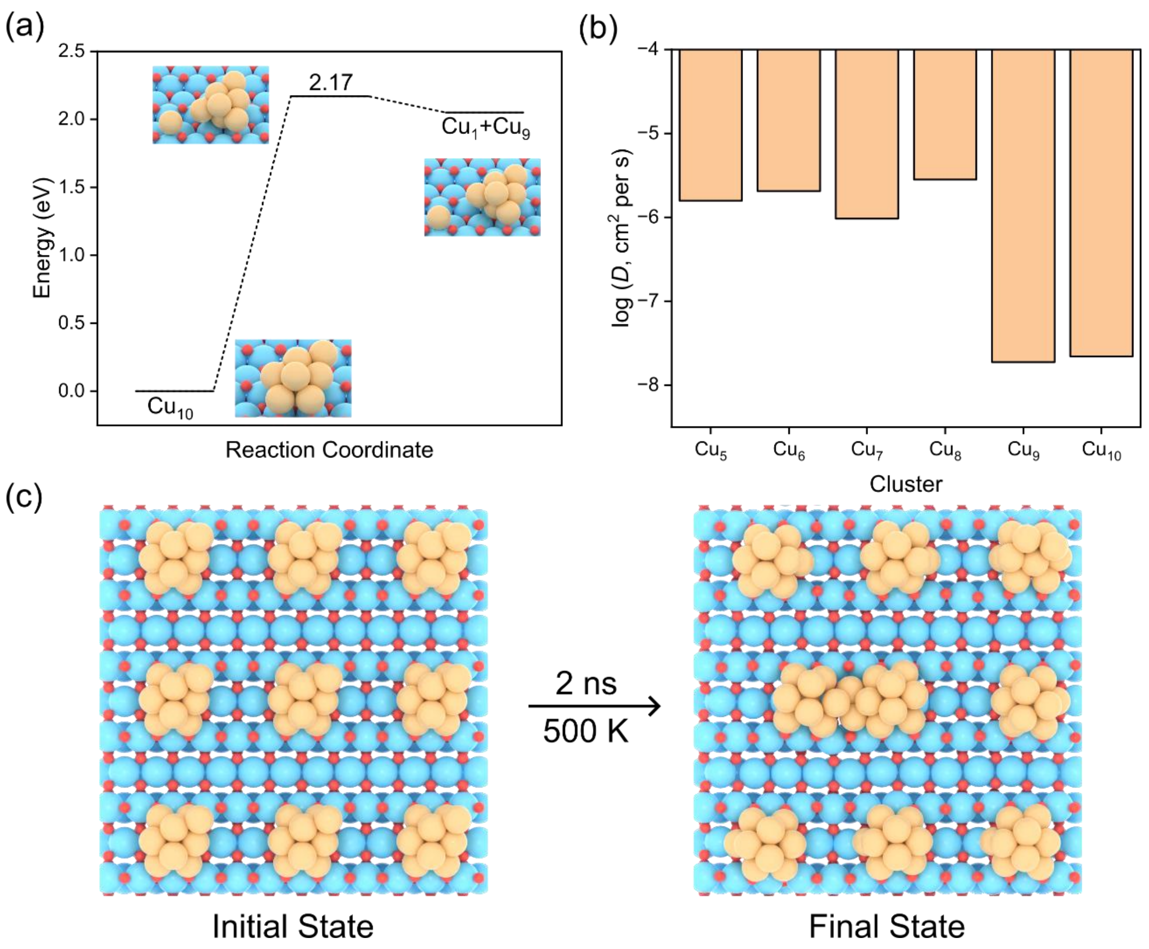

Supplementary Figure 7. Fragmentation and sintering dynamics of Cu clusters on TiO_2. (a) Minimum energy path for the fragmentation of a Cu_{10} cluster into Cu_1 and Cu_9 on the TiO_2 surface. The activation barrier (2.17 eV) for the rate-determining step is highlighted. (b) Size-dependent diffusion coefficients (*D*) for various Cu clusters (Cu_5–Cu_{10}) plotted on a logarithmic scale. The unit is cm^2/s. (c) Representative snapshots from neural network (NN) potential-based molecular dynamics (MD) simulations illustrating the sintering process at 500 K over a 2 ns trajectory.

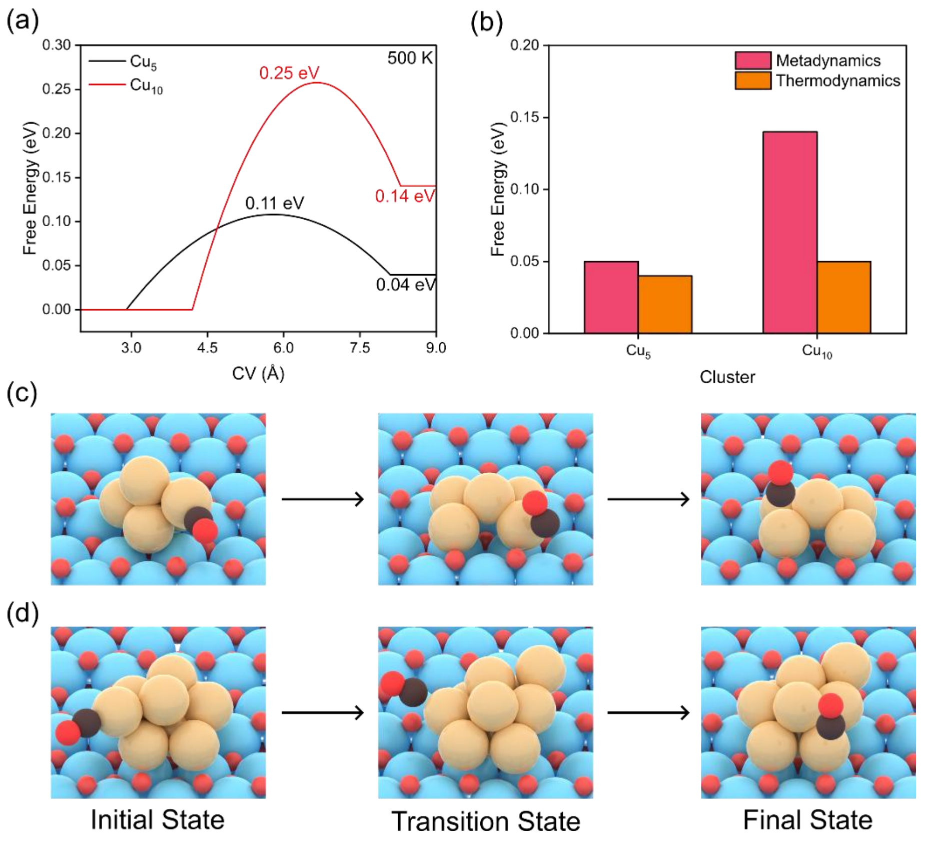

Supplementary Figure 8. Free energy landscapes and structural evolution during CO desorption. (a) Free energy profiles of CO desorption from Cu_5 and Cu_{10} clusters at 500 K as a function of the collective variable (CV). The CV is defined as the distance between the Cu cluster center of mass and the carbon atom, which is sampled via 1 ns NNP-based molecular dynamics simulations. (b) Comparative desorption free energies obtained from enhanced sampling versus static thermodynamic calculations for Cu_5 and Cu_{10} at 500 K. (c, d) Representative snapshots illustrating the initial, transition, and final states of the desorption process for (c) Cu_5 and (d) Cu_{10}. Color scheme: Ti (blue), O (red), Cu (gold), and C (black).

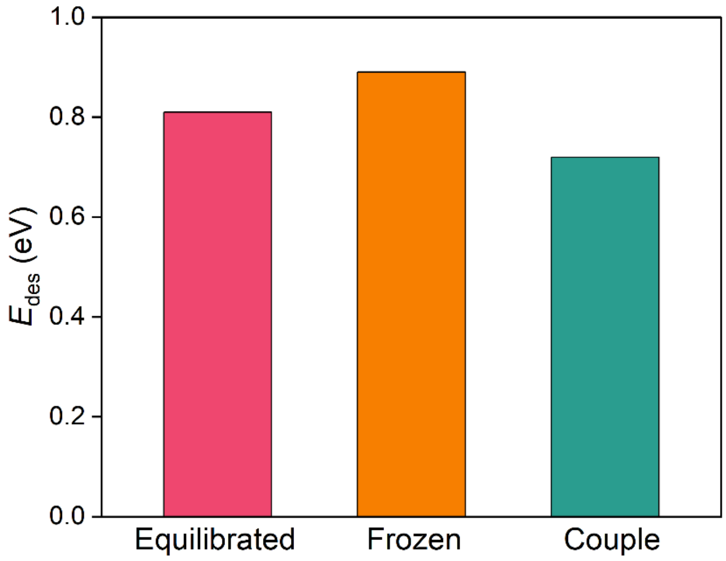

Supplementary Figure 9. CO desorption energy vs N_c ratio for Cu_7/TiO_2. The bar chart shows the CO desorption energy (E_{des}, eV) with varying values of N_c. The red bar represents the fast-structure limit ($N_c < 10^{-4}$), the orange bar corresponds to the coupled regime ($N_c \sim 1$), and the teal bar represents the frozen regime ($N_c > 10^4$), where the rate gain decreases.

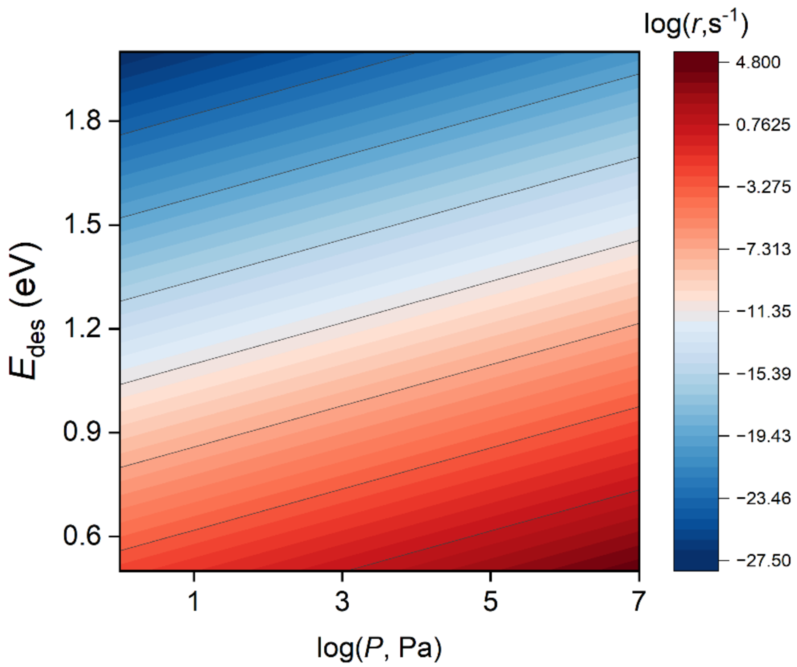

Supplementary Figure 10. Pressure–desorption-energy map of the CO adsorption/desorption rate. 2D contour plot of rate (*r*) as a function of desorption energy E_{des} and gas pressure *P* at 300 K. Warmer colors indicate faster rates. The nearly linear iso-rate lines (gray) show that *r* increases with *P* and decreases with E_{des}. The map delineates the transition from a desorption-limited regime (blue; high E_{des}, low *P*) to facile desorption (red; low E_{des}, high *P*). The units of *r*, E_{des} and *P* are s^{-1}, eV and Pa, respectively.

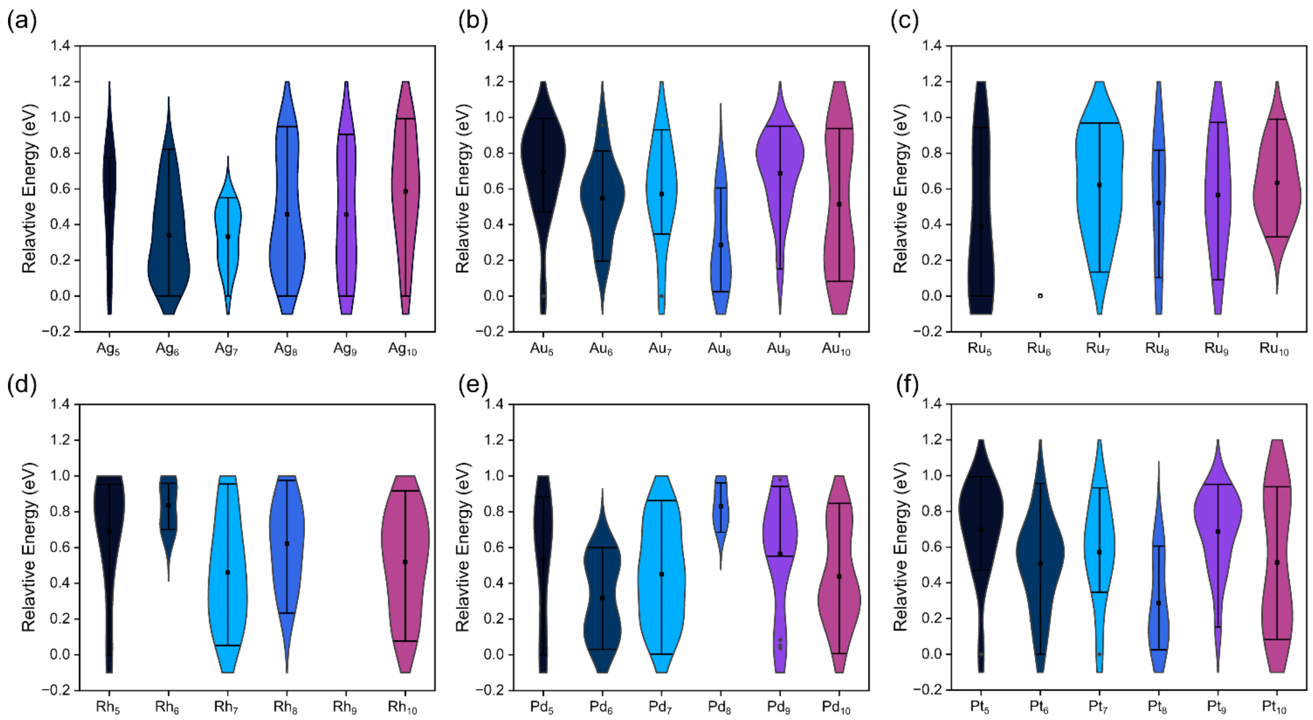

Supplementary Figure 11. Property distributions of M_n/TiO_2 clusters across structural ensembles. Violin plots showing the distribution of relative energies for different M_n (M = Ag, Au, Ru, Rh, Pd, Pt; n = 5–10) clusters on TiO_2(110), sampled from multiple local minima with distinct geometries. Each panel corresponds to a distinct structural motif or cluster. The colored violins represent the full ensemble of accessible states, whereas the black markers denote the values associated with the lowest-energy configuration for each case.

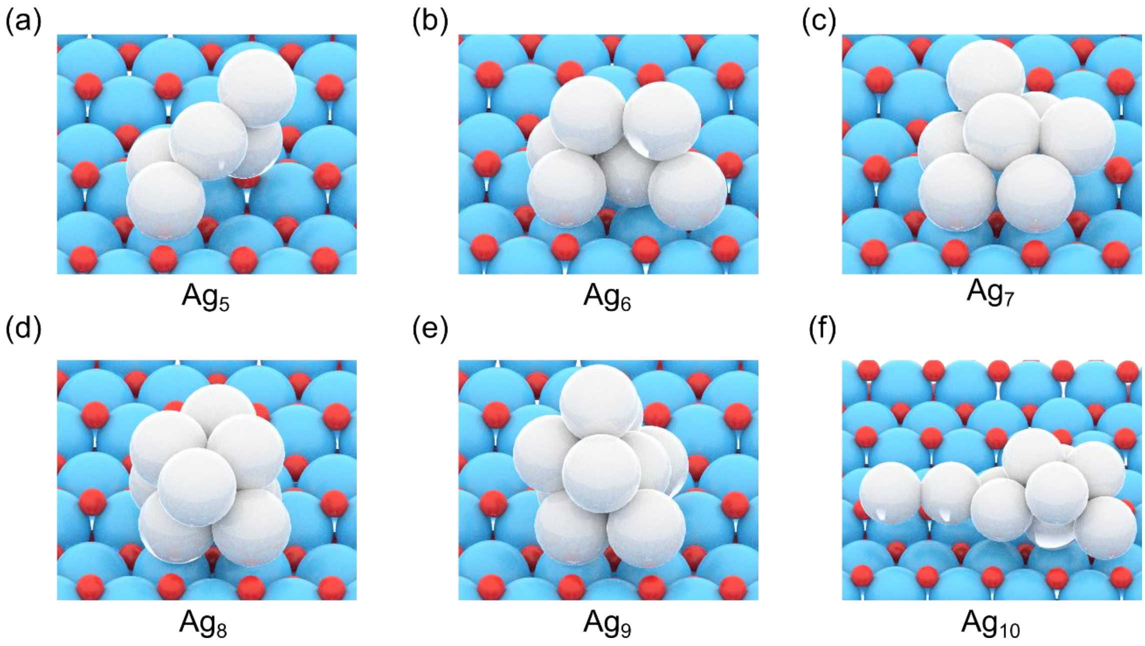

Supplementary Figure 12. Representative Ag_n/TiO_2 structures with increasing clusters (n = 5–10). Configurations with varying numbers of Ag clusters supported on TiO_2(110). The white spheres denote Ag atoms.

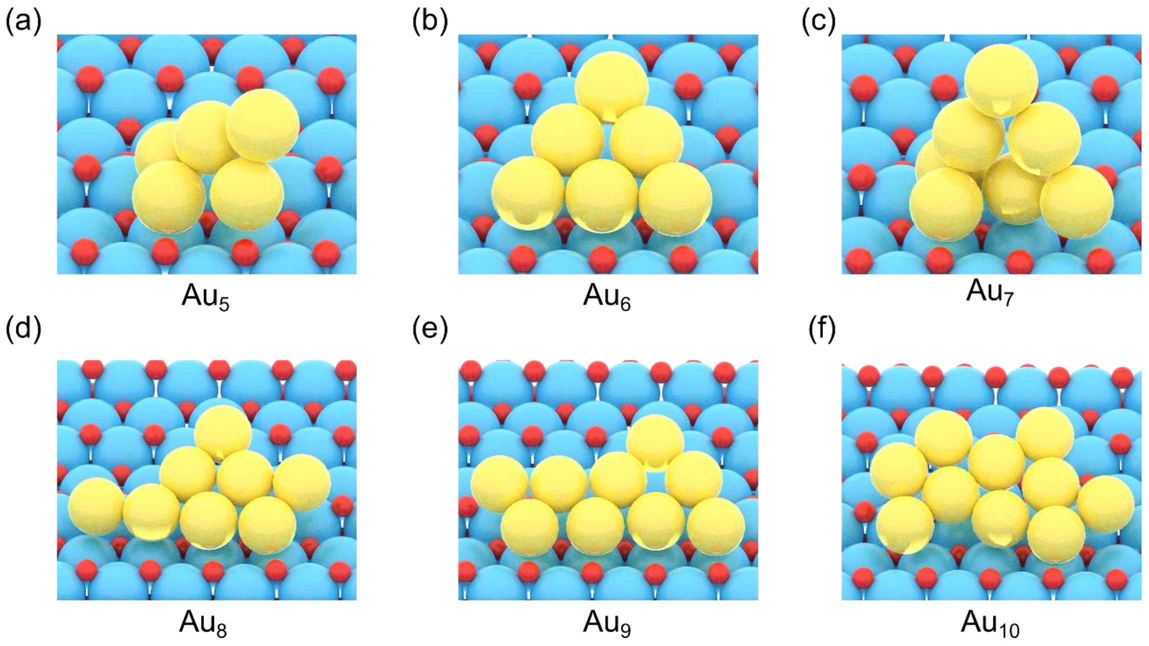

Supplementary Figure 13. Representative Au_n/TiO_2 structures with increasing clusters (n = 5–10). Configurations with varying numbers of Au clusters supported on TiO_2(110). Yellow spheres denote Au atoms.

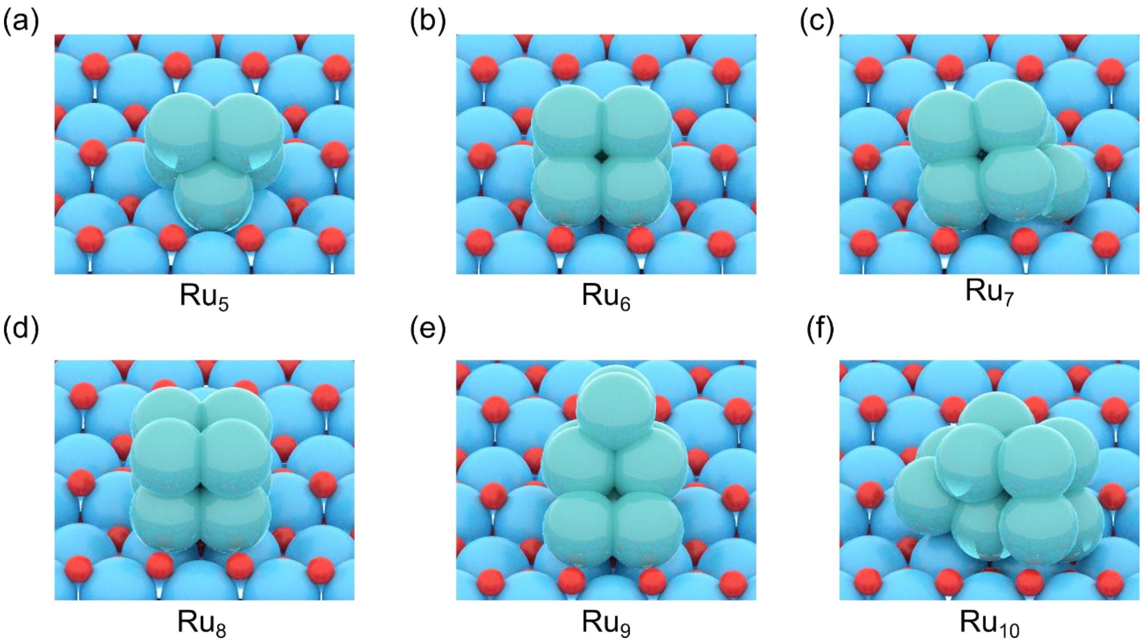

Supplementary Figure 14. Representative Ru_n/TiO_2 structures with increasing clusters (n = 5–10). Configurations with varying numbers of Ru clusters supported on TiO_2(110). The green spheres denote Ru atoms.

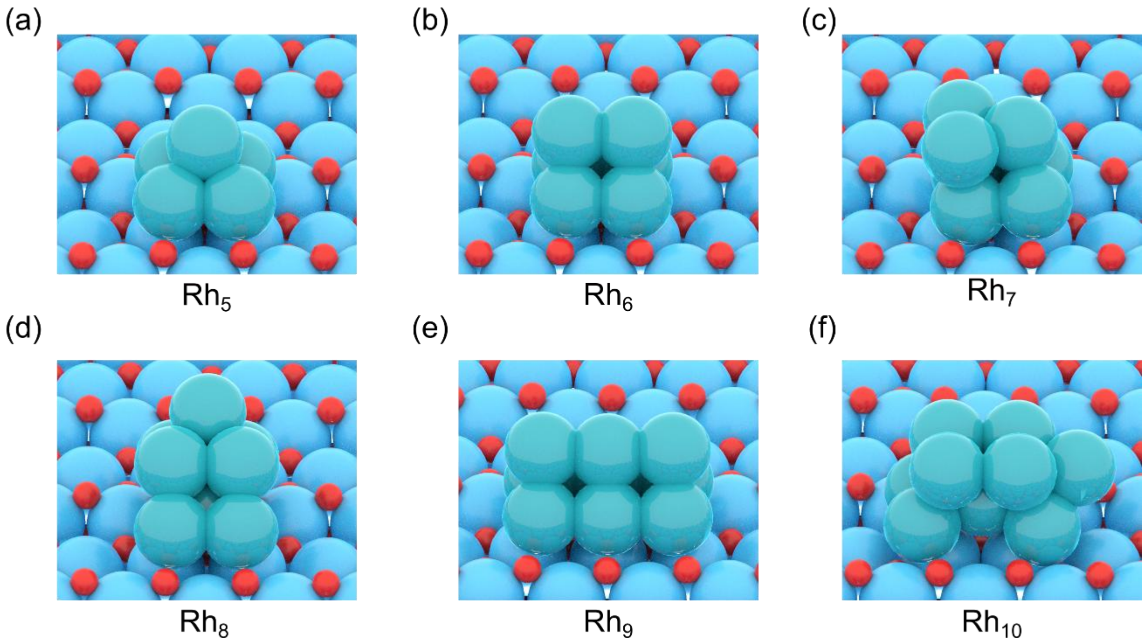

Supplementary Figure 15. Representative Rh_n/TiO_2 structures with increasing clusters (n = 5–10). Configurations with varying numbers of Rh clusters supported on $TiO_2(110)$. The green spheres denote Rh atoms.

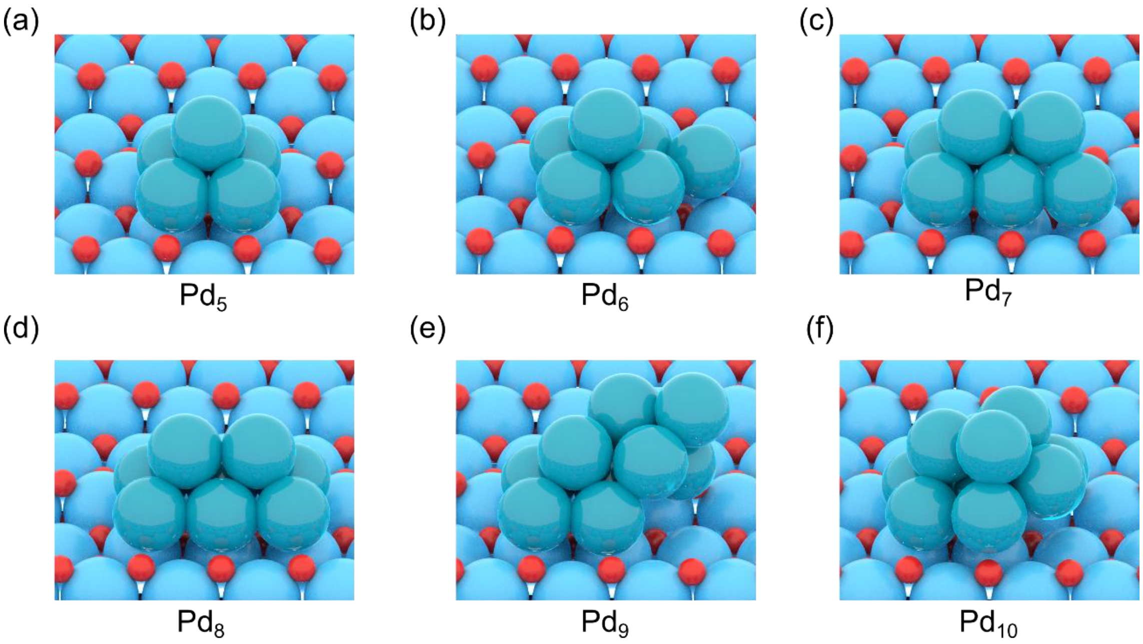

Supplementary Figure 16. Representative Pd_n/TiO_2 structures with increasing clusters (n = 5–10). Configurations with varying numbers of Pd clusters supported on TiO_2(110). The green spheres denote Pd atoms.

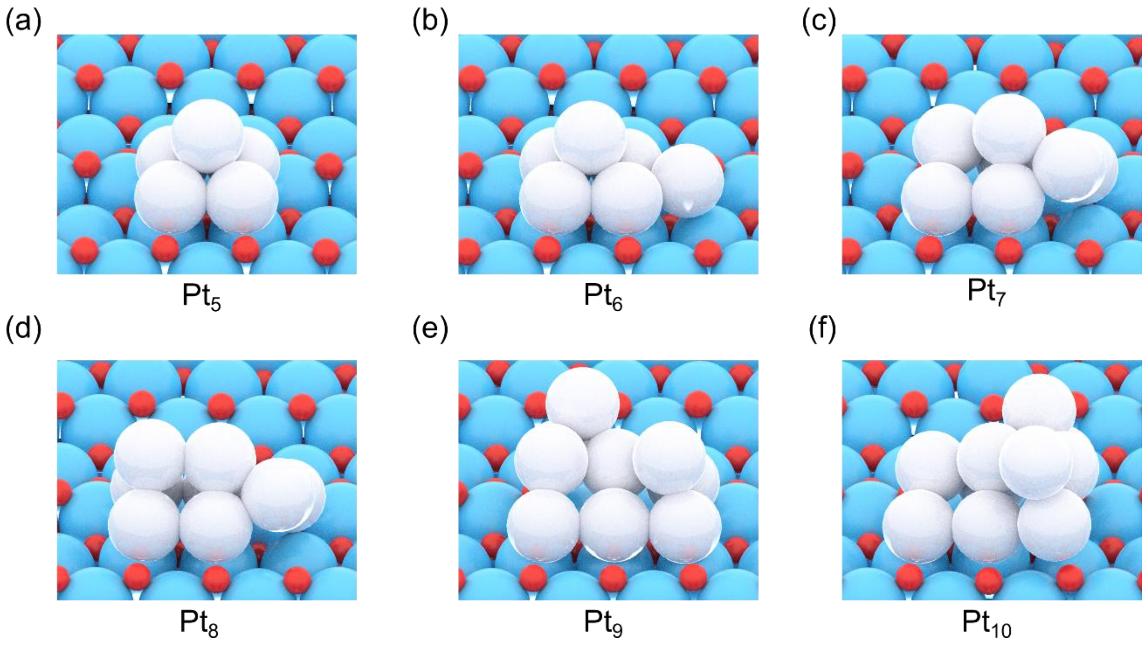

Supplementary Figure 17. Representative Pt_n/TiO_2 structures with increasing clusters (n = 5–10). Configurations with varying numbers of Pt clusters supported on TiO_2(110). The white spheres denote Pt atoms.

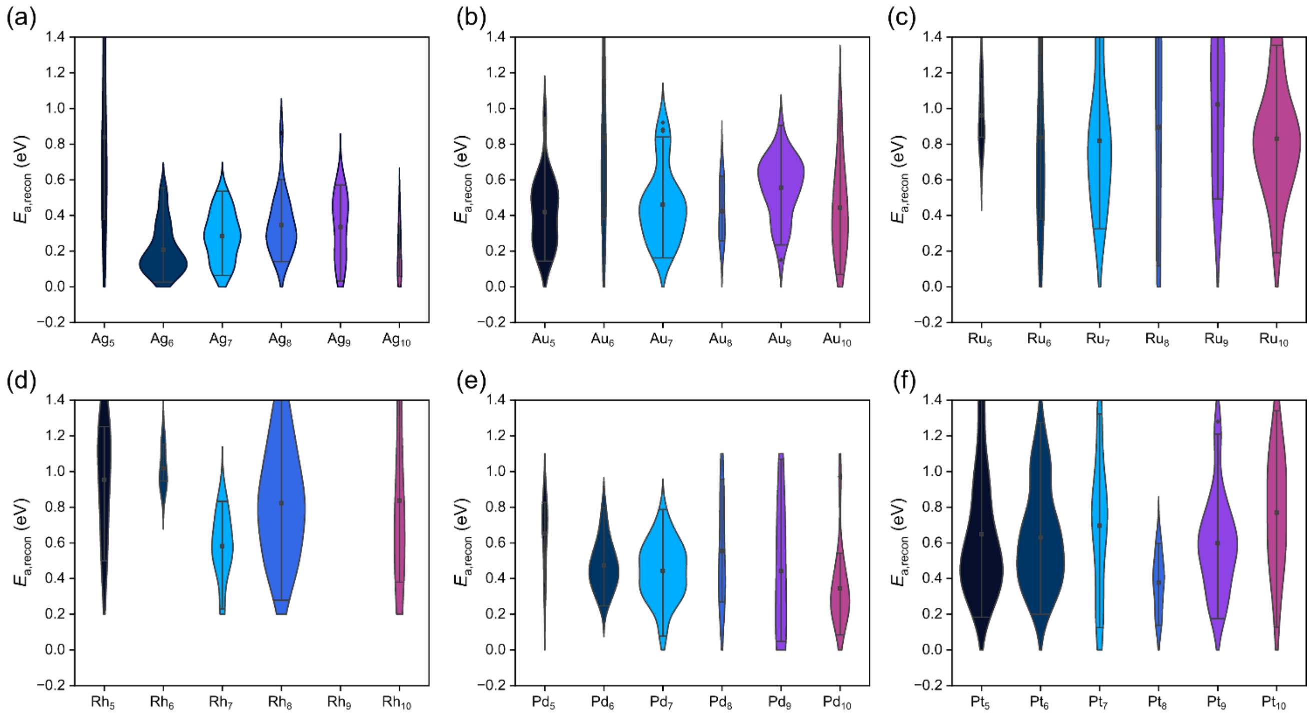

Supplementary Figure 18. Distribution of forward reconstruction barriers ($E_{a,\ recon}$) for M_n/TiO_2 clusters across structural ensembles. Violin plots illustrate the statistical spread of forward transition-state energies associated with structural reconstruction events for different M_n (M = Ag, Au, Ru, Rh, Pd, Pt; n = 5–10) clusters on TiO_2(110). Each panel corresponds to a distinct structural motif or cluster. The results reveal cluster size-dependent variations in both the median and distribution width of $E_{ts,recon}$, reflecting structural fluxionality and the configurational complexity of the potential energy landscape.

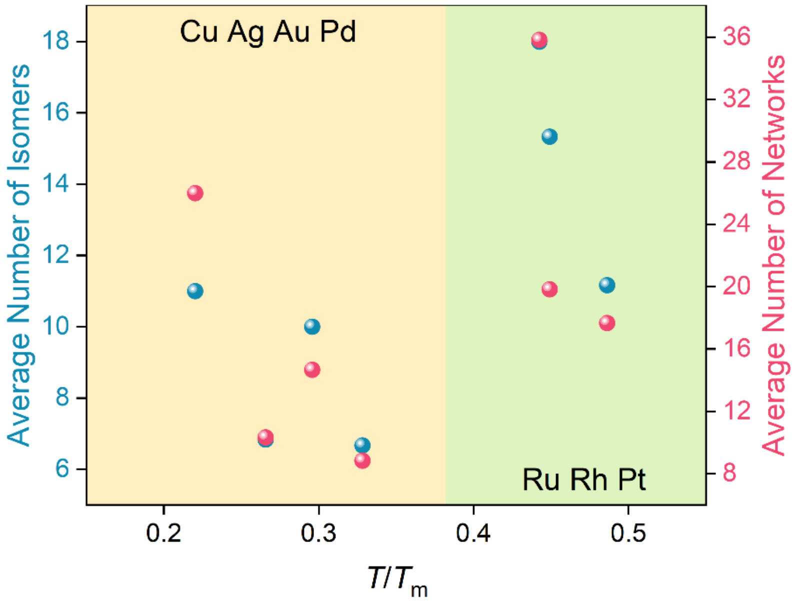

Supplementary Figure 19. Correlation between the average number of isomers and networks and the ratio of the reaction temperature (T = 300 K) and bulk Tammann temperature (T/T_m) of the metal clusters. The plot shows that as the Tammann temperature increases, both the average number of isomers (left axis, blue markers) and the average number of networks (right axis, pink markers) differ.

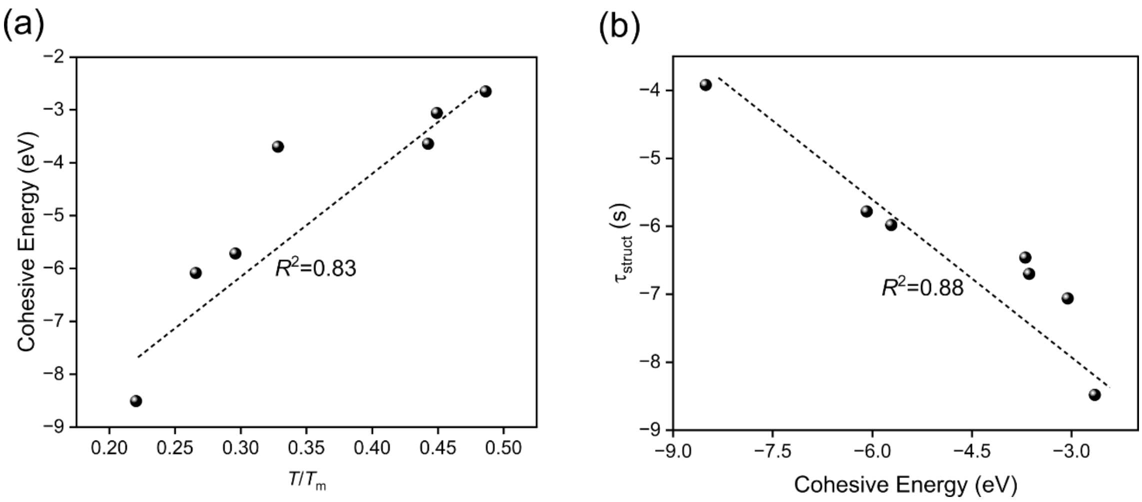

Supplementary Figure 20. Scaling relationships between cohesive energy, Tammann temperature, and structural reconstruction. (a) Linear correlation between cohesive energy and the reduced Tammann temperature (T/T_m). The robust linear dependency (R^2 = 0.83) identifies cohesive energy as a primary descriptor of the thermal stability of metal clusters. (b) Relationship between the cohesive energy and the structural reconstruction time (τ_{struct}) at 300 K. The strong linear scaling (R^2 = 0.88) across various cluster species demonstrates that the kinetic lifetime of the subnanometer catalysts is intrinsically governed by their cohesive strength.

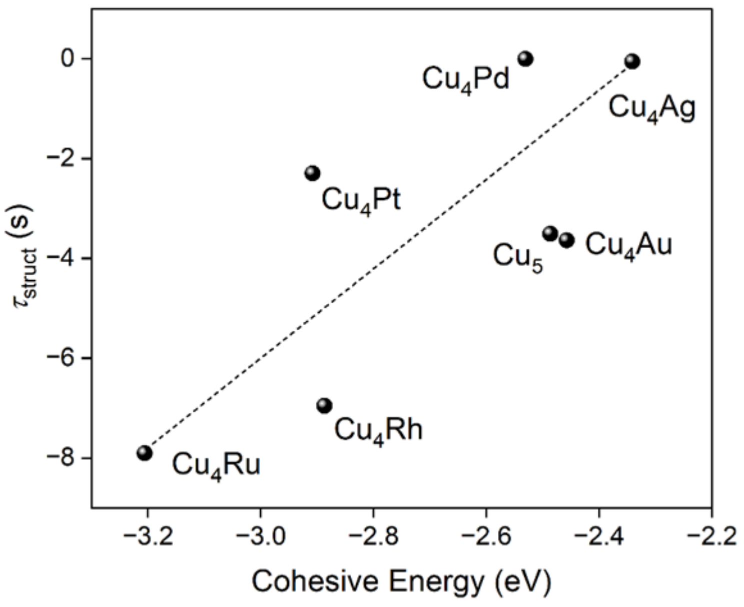

Supplementary Figure 21. Correlation between the cohesive energy and structural stability of Cu-based single-atom alloys (SAAs). The structural reconstruction time (τ_{struct}) of the Cu_4M_1 clusters supported on TiO_2 scales linearly with their calculated cohesive energies. d

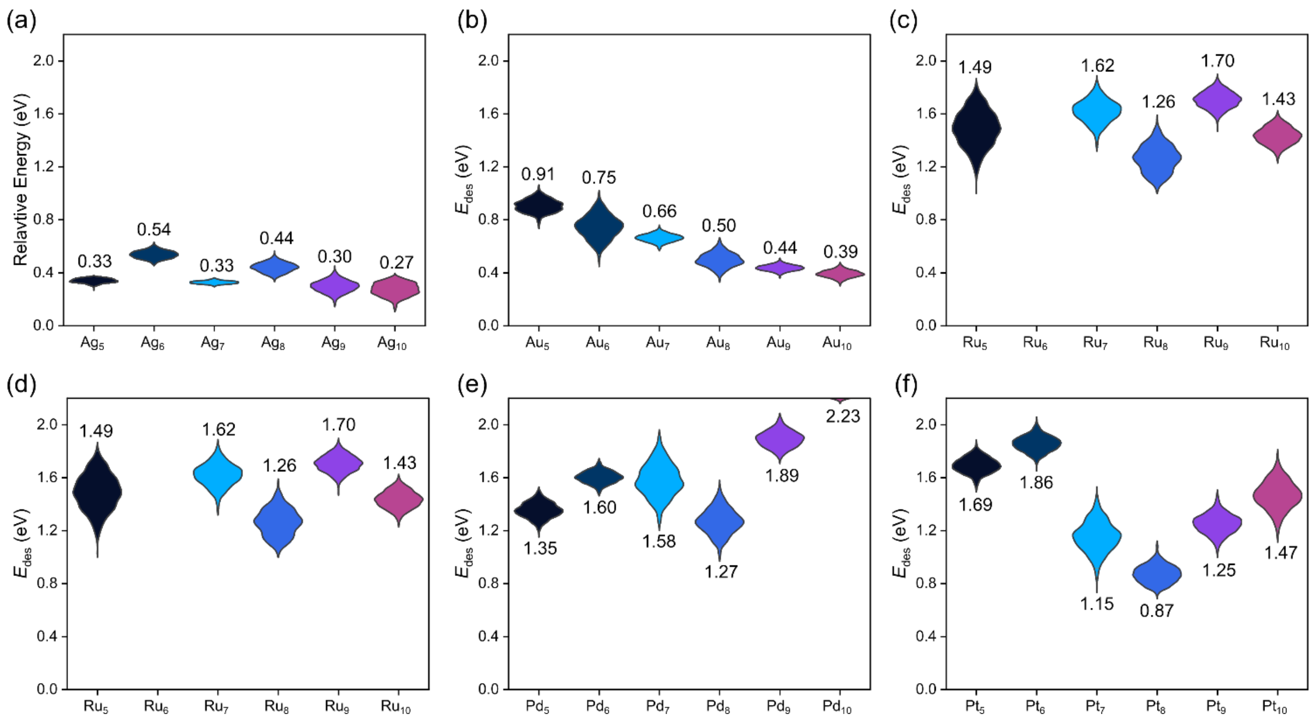

Supplementary Figure 22. Distribution of CO desorption energies on M_n/TiO_2 clusters across structural ensembles. Violin plots showing the distribution of CO desorption energies (E_{des}) sampled from thermally accessible configurations of different M_n (M = Ag, Au, Ru, Rh, Pd, Pt; n = 5–10) clusters on TiO_2(110).

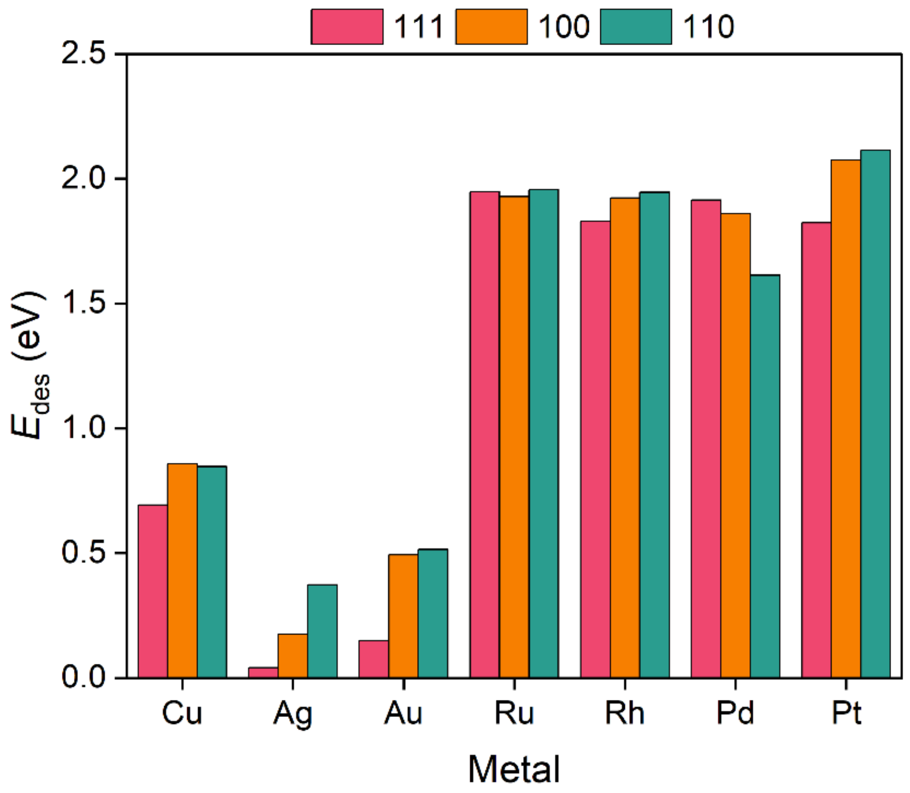

Supplementary Figure 23. CO desorption energies on low-index facets of transition metals. Calculated CO desorption energies (E_{des}) on the (111), (100), and (110) surfaces of seven late transition metals: Cu, Ag, Au, Ru, Rh, Pd, and Pt. Each bar represents the desorption energy on a specific facet, color-coded as indicated in the legend.

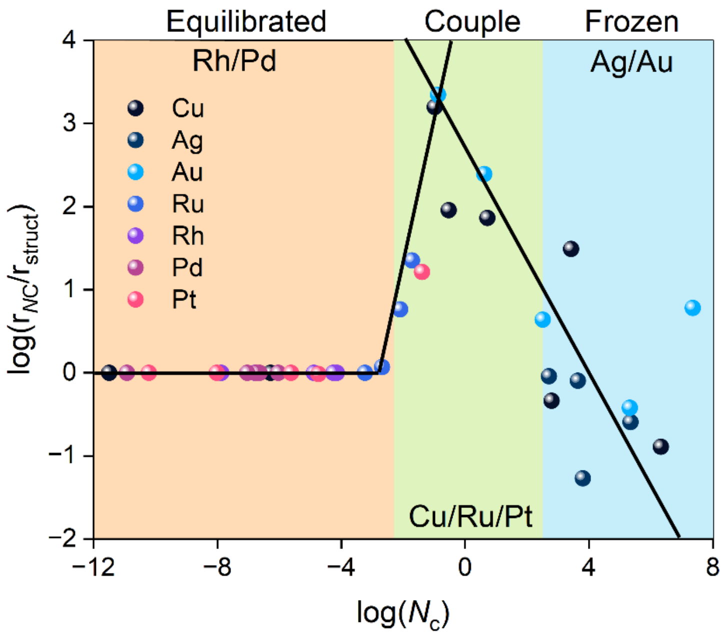

Supplementary Figure 24. Rate enhancement vs. the N_c ratio for M_n/TiO_2. Scatter plot of $G=r_{Nc}/r$ as a function of N_c, where $N_c=\tau_{struct}/\tau_{chem}$. Each point corresponds to a cluster size (M_5–M_{10}, M: Cu, Ag, Au, Ru, Rh, Pd, Pt). Three regimes emerge: for $N_c < 10^{-4}$ (orange, fast-structure limit), the enhancement is negligible; for $N_c > 10^{-3}$ and $N_c < 10^3$ (green, structure–reaction couple regime), the enhancement with N_c in the volcano plot; and for $N_c > 10^3$ (blue, reaction regime), the improvement is less than the most stable state.

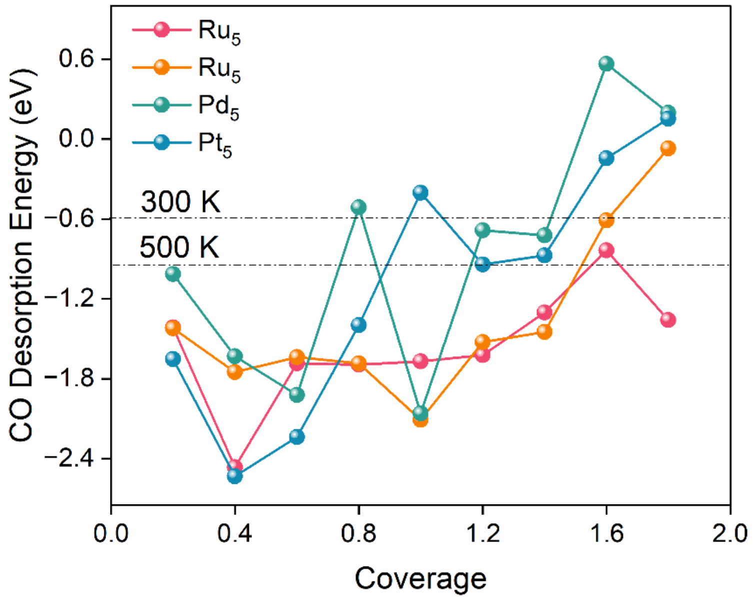

Supplementary Figure 25. CO desorption energies as a function of surface coverage from 0 to 2. Calculated CO desorption energies (E_{des}) on Ru, Rh, Pd, and Pt surfaces as a function of CO coverage. While the absolute desorption energies vary across metals, all systems generally exhibit a decreasing trend in E_{des} with increasing coverage, reflecting increasing adsorbate–adsorbate repulsion.

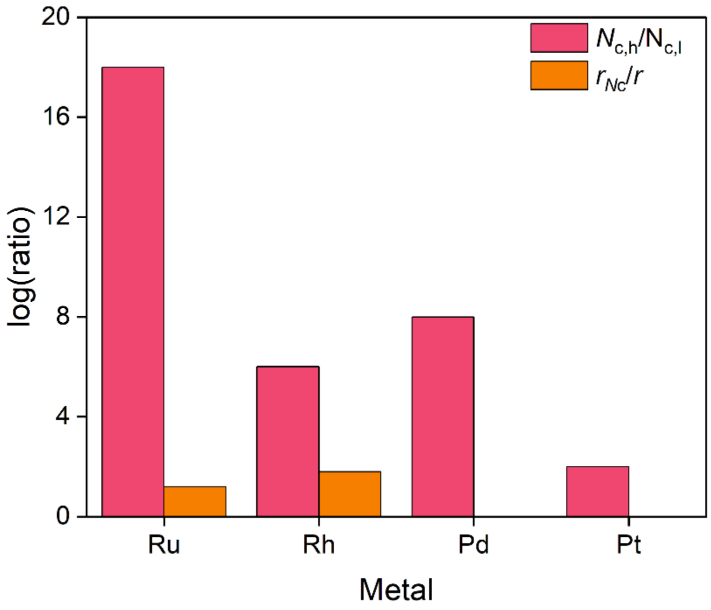

Supplementary Figure 26. Metal-dependent shifts in the time–scale ratio and rate gain. Bar charts of log($N_{c,h}$ /$N_{c,l}$) (pink) and log(r_{Nc}/r) (orange) for Ru, Rh, Pd, and Pt. $N_{c,h}$ and $N_{c,l}$ are Nc with high and low coverage. Metals that drive N_c toward the gating regime (N_c~1) exhibit greater rate amplification: Ru and Rh clearly gain, whereas Pd (shift remaining in the fast-structure limit) and Pt (minimal shift) yield negligible r_{Nc}/r values. The y-axis is the base-10 logarithm; the ratios are dimensionless.

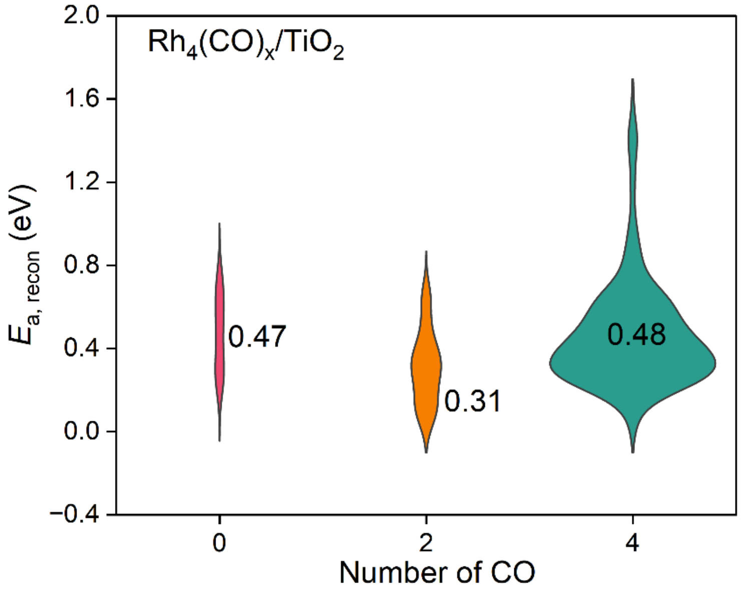

Supplementary Figure 27. Structural reconstruction forward transition state energies of $Rh_4(CO)_x/TiO_2$ as a function of CO coverage. The calculated reconstruction energies ($E_{a,recon}$) for $Rh_4(CO)_x/TiO_2$ clusters with varying CO coverages are presented. As the number of CO molecules increases from 0 to 4, the reconstruction energy significantly increases, with the average E_a increasing from 0.47 eV for $x = 0$ (coverage = 0) to 0.48 eV for $x = 4$ (coverage = 1).

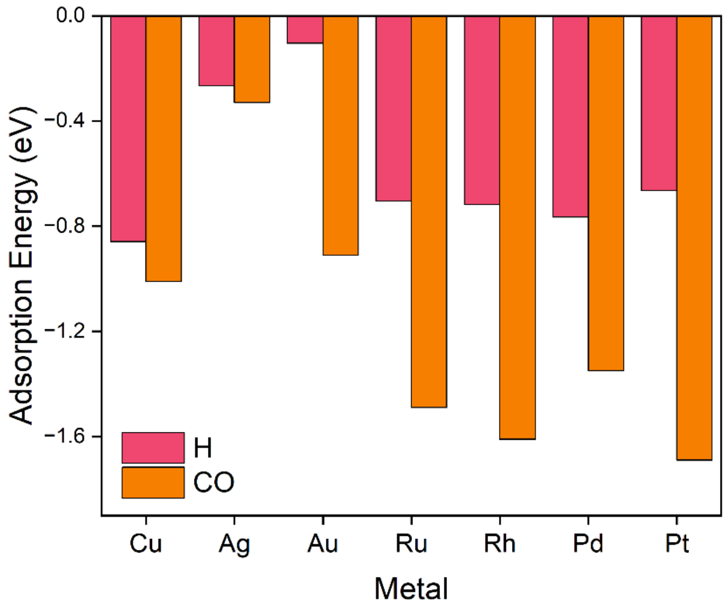

Supplementary Figure 28. Hydrogen adsorption energies for different metals. Calculated CO and H adsorption energies for 5 atoms of Cu, Ag, Au, Ru, Rh, Pd, and Pt clusters supported on TiO_2(110). The clusters are the most stable structures.

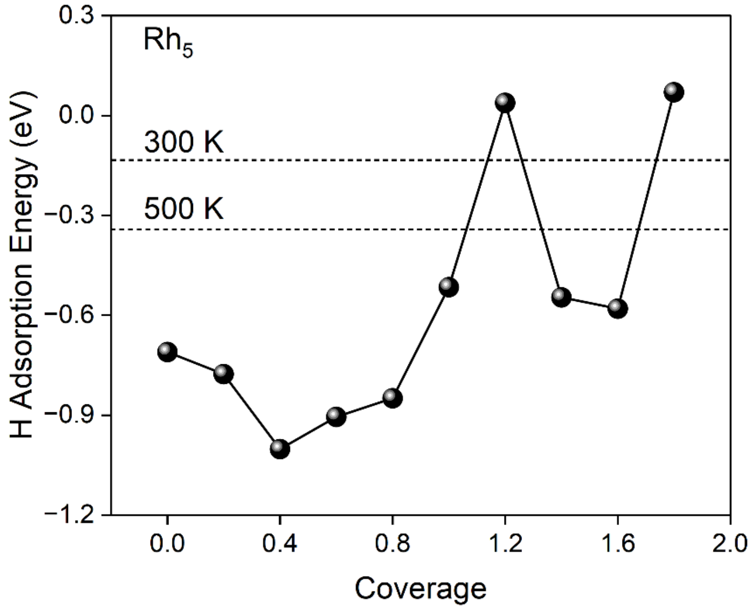

Supplementary Figure 29. Hydrogen adsorption energies with different CO coverages. Calculated hydrogen adsorption energies for different CO coverages from 0 to 2 on Rh clusters supported on TiO_2(110).

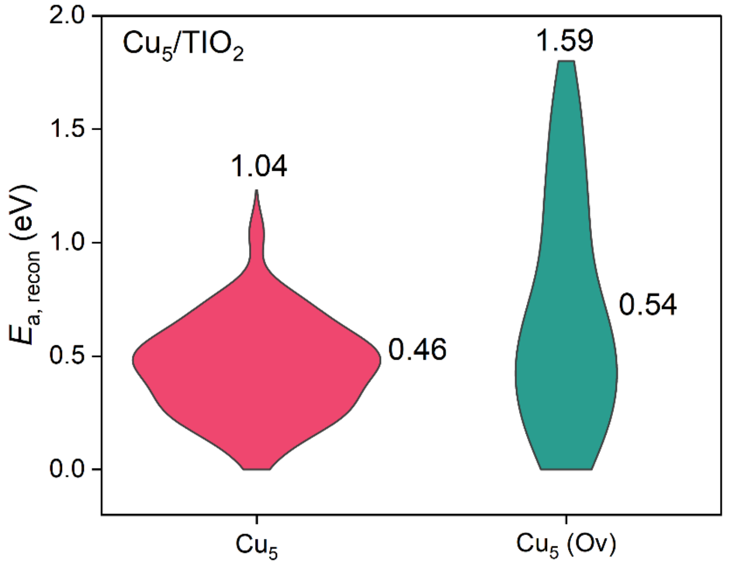

Supplementary Figure 30. Structural reconstruction forward transition state energies for Cu_5/TiO_2 clusters with and without TiO_2 oxygen vacancies with structures at 1 eV. The calculated reconstruction energies ($E_{a,recon}$) for the Cu_5 clusters with and without oxygen vacancies (Cu_5 (Ov)) are shown. The addition of oxygen vacancies results in a higher $E_{a,recon}$, with Cu_5 (Ov) exhibiting a wider energy distribution than the pristine Cu_5 cluster does, indicating greater structural flexibility when oxygen vacancies are present. The maximum and median $E_{a,recon}$ values are 1.04 and 0.46 eV for Cu_5 and 1.59 and 0.54 eV for Cu_5 (Ov), respectively, suggesting that the presence of oxygen vacancies significantly expands the time of structural equilibrium.

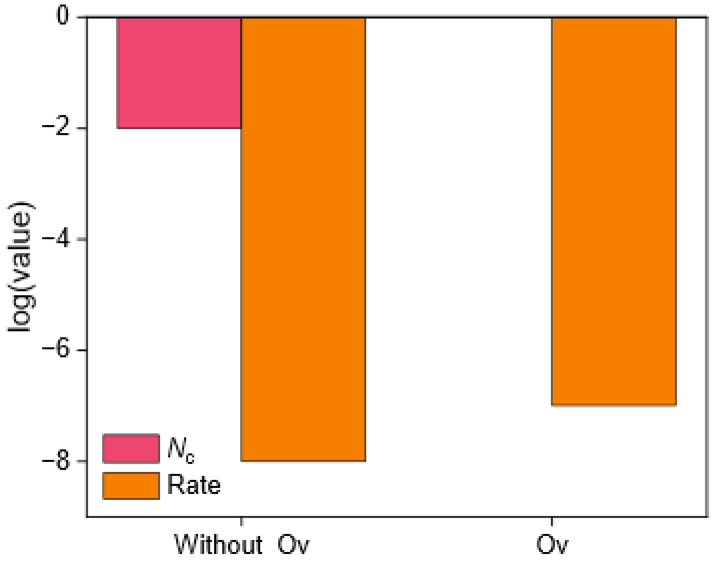

Supplementary Figure 31. Effect of oxygen vacancies on the time–scale ratio and rate. The bars represent N_c (pink) and r (orange) for surfaces without and with oxygen vacancies (Ov). The vacancy-free case has N_c~10^{-2} (fast-structure limit) and $r\sim10^{-8}$ s^{-1}; introducing Ov further lowers the absolute rate to $r\sim10^{-7}$ s^{-1} while keeping N_c in the same fast-structure regime (below the plotted range). Thus, Ov primarily reduces the baseline kinetics rather than pushing the system toward the gating region, yielding little coupling gain.

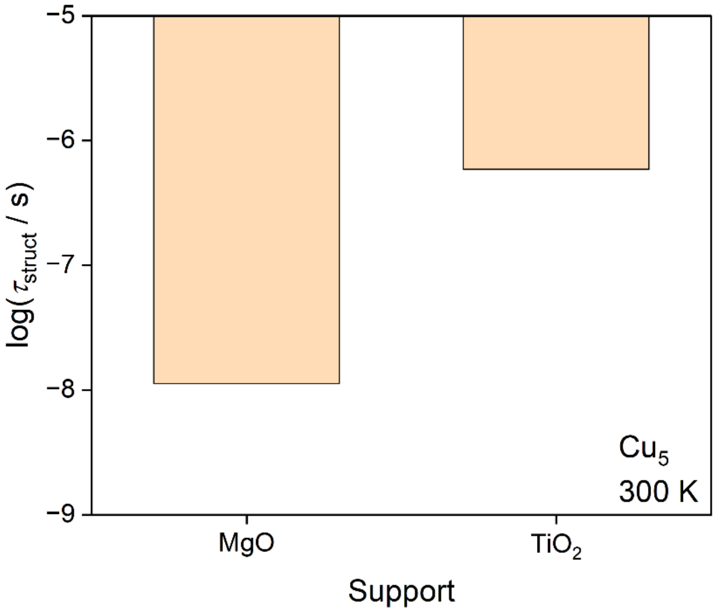

Supplementary Figure 32. The difference in τ_{struct} between MgO and TiO_2. We employed a Cu_5 cluster supported on different metal oxidations at 300 K.

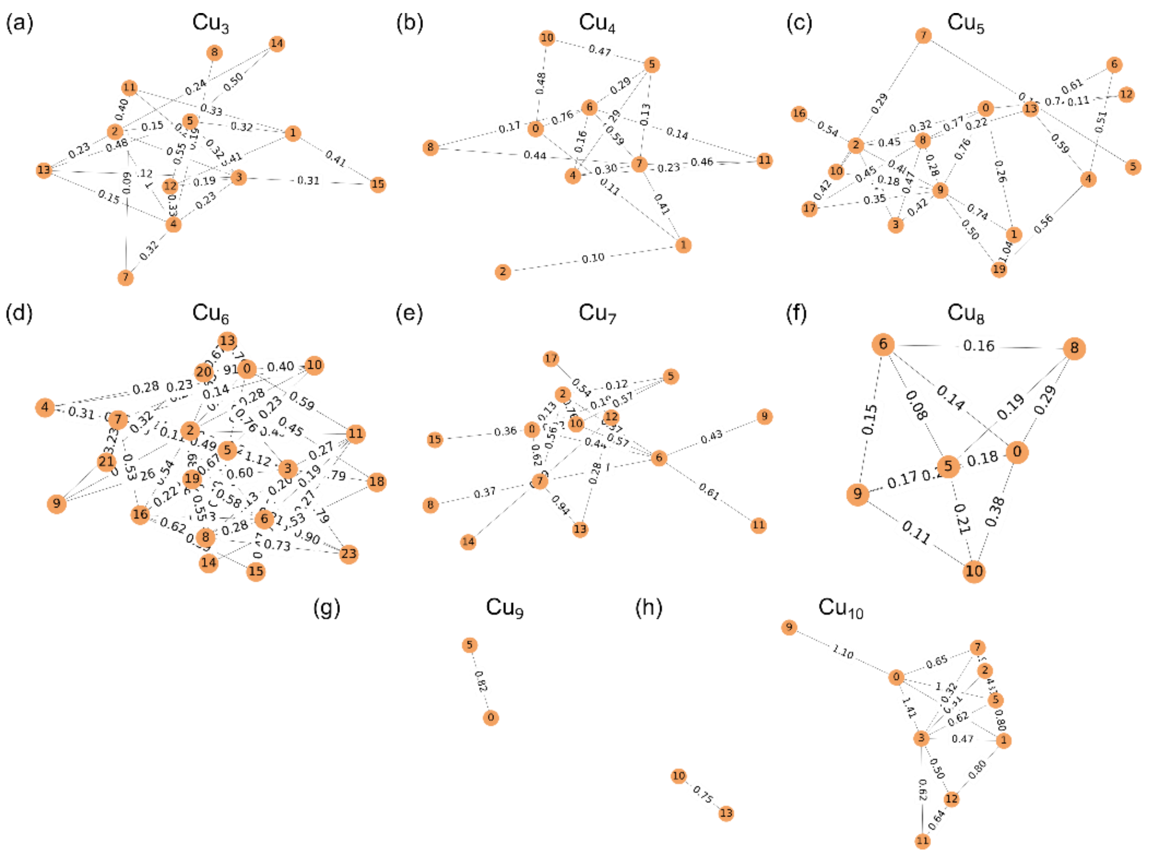

Supplementary Figure 33. The isomer networks over different Cu clusters (Cu_3 to Cu_{10}) supported on TiO_2. Isomerization pathways are represented as graph networks where nodes denote specific structural isomers and edges indicate elementary transformation steps. Numerical labels on edges quantify the forward activation barriers from stable to metastable configurations, expressed in eV.

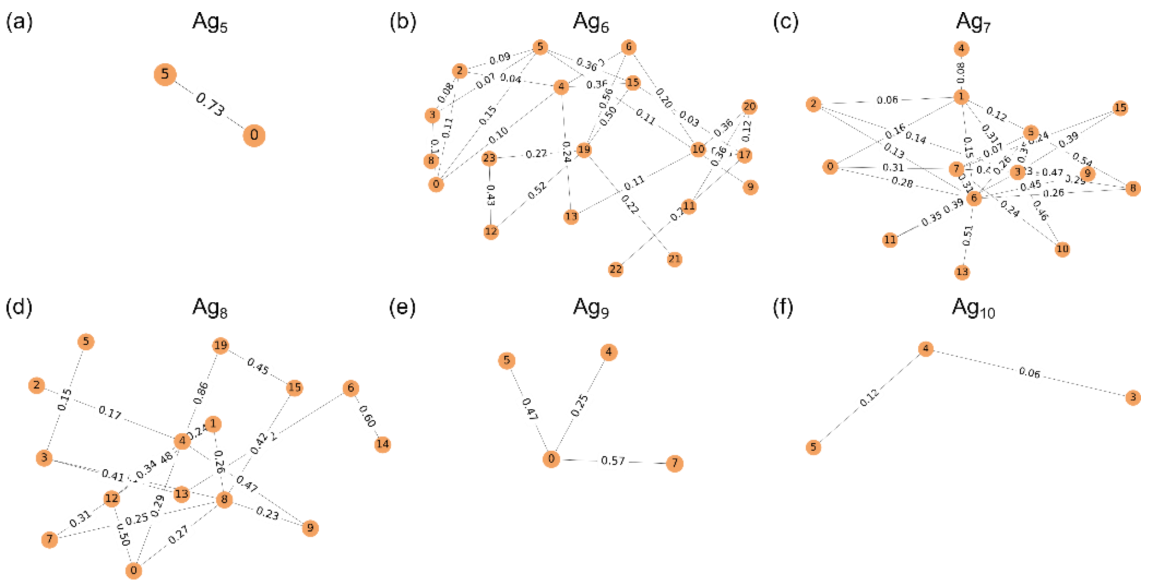

Supplementary Figure 34. The isomer networks over different Ag cluster (Ag_5 to Ag_{10}) supported on TiO_2. Isomerization pathways are represented as graph networks where nodes denote specific structural isomers and edges indicate elementary transformation steps. Numerical labels on edges quantify the forward activation barriers from stable to metastable configurations, expressed in eV.

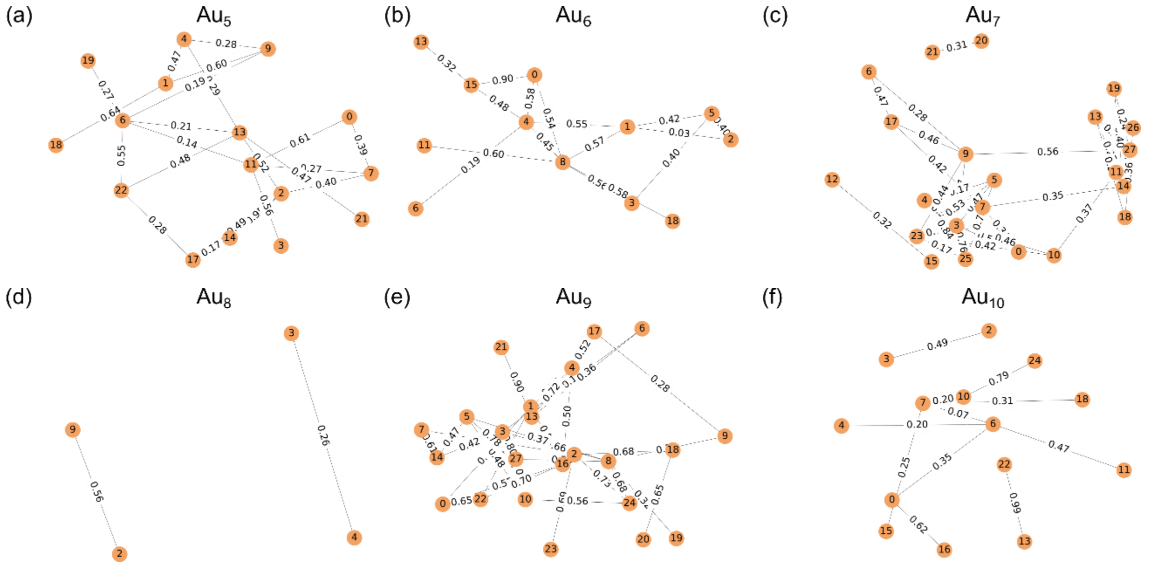

Supplementary Figure 35. The isomer networks over different Au cluster (Au_5 to Au_{10}) supported on TiO_2. Isomerization pathways are represented as graph networks where nodes denote specific structural isomers and edges indicate elementary transformation steps. Numerical labels on edges quantify the forward activation barriers from stable to metastable configurations, expressed in eV.

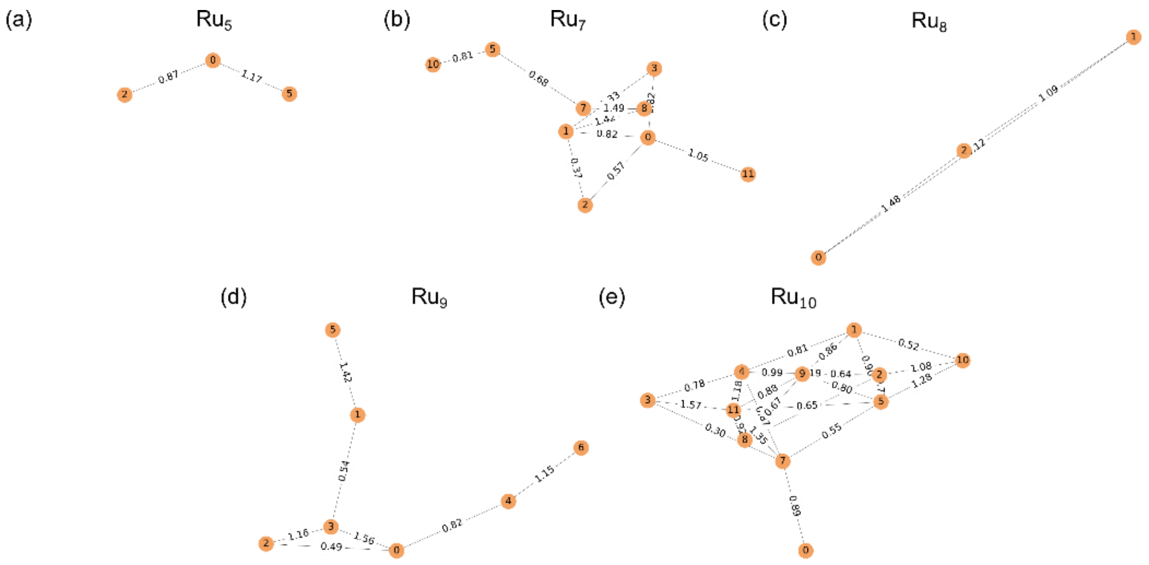

Supplementary Figure 36. The isomer networks over different Ru cluster (Ru_5 to Ru_{10}) supported on TiO_2. Isomerization pathways are represented as graph networks where nodes denote specific structural isomers and edges indicate elementary transformation steps. Numerical labels on edges quantify the forward activation barriers from stable to metastable configurations, expressed in eV.

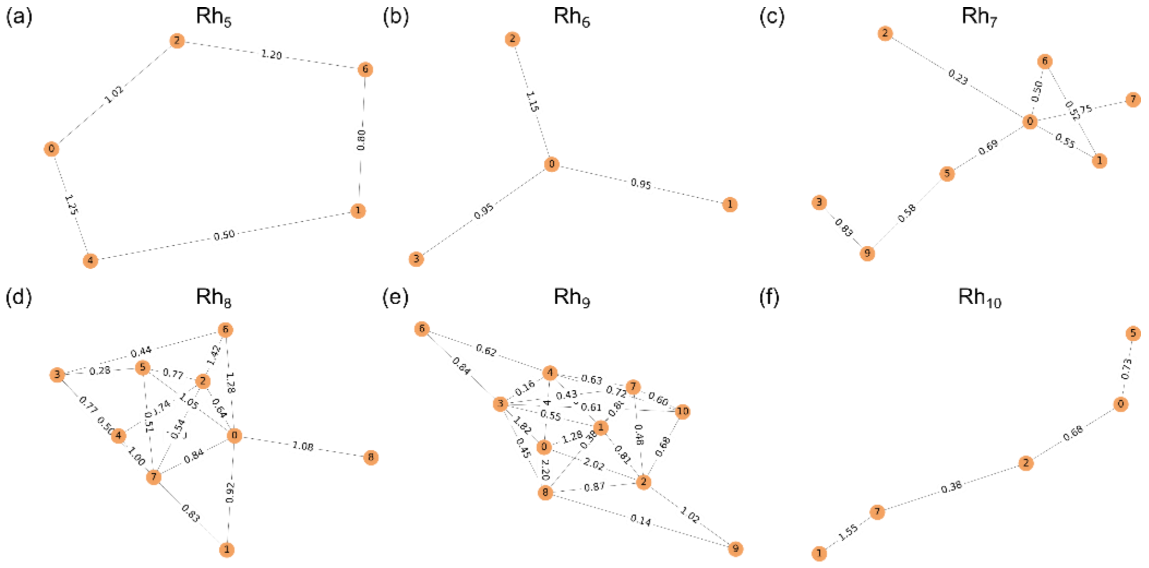

Supplementary Figure 37. The isomer networks over different Rh cluster (Rh_5 to Rh_{10}) supported on TiO_2. Isomerization pathways are represented as graph networks where nodes denote specific structural isomers and edges indicate elementary transformation steps. Numerical labels on edges quantify the forward activation barriers from stable to metastable configurations, expressed in eV.

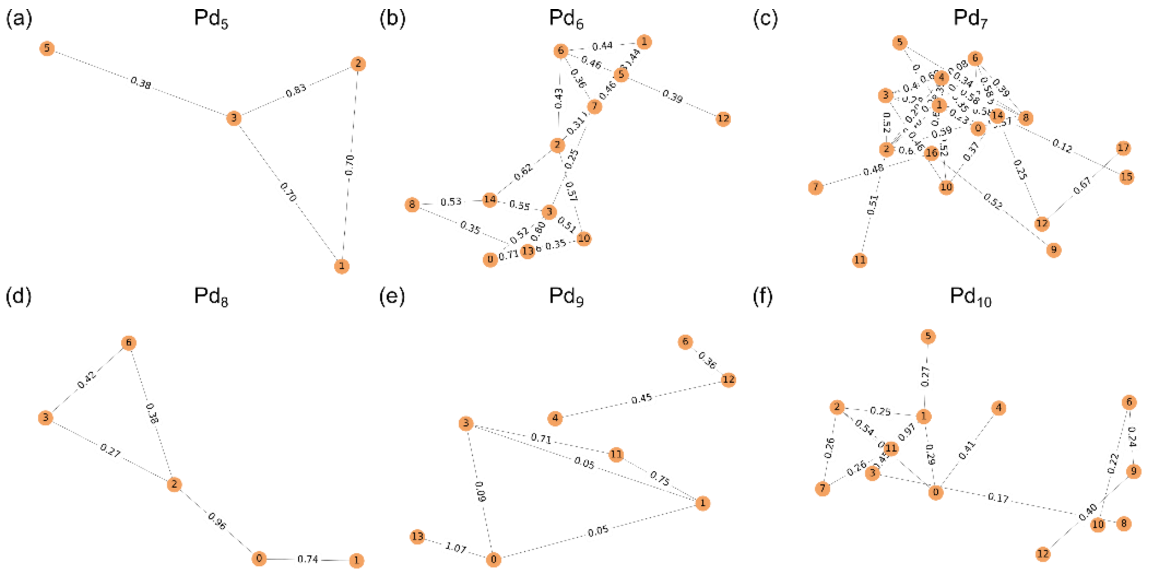

Supplementary Figure 38. The isomer networks over different Pd cluster (Pd_5 to Pd_{10}) supported on TiO_2. The Isomerization pathways are represented as graph networks where nodes denote specific structural isomers and edges indicate elementary transformation steps. Numerical labels on edges quantify the forward activation barriers from stable to metastable configurations, expressed in eV.

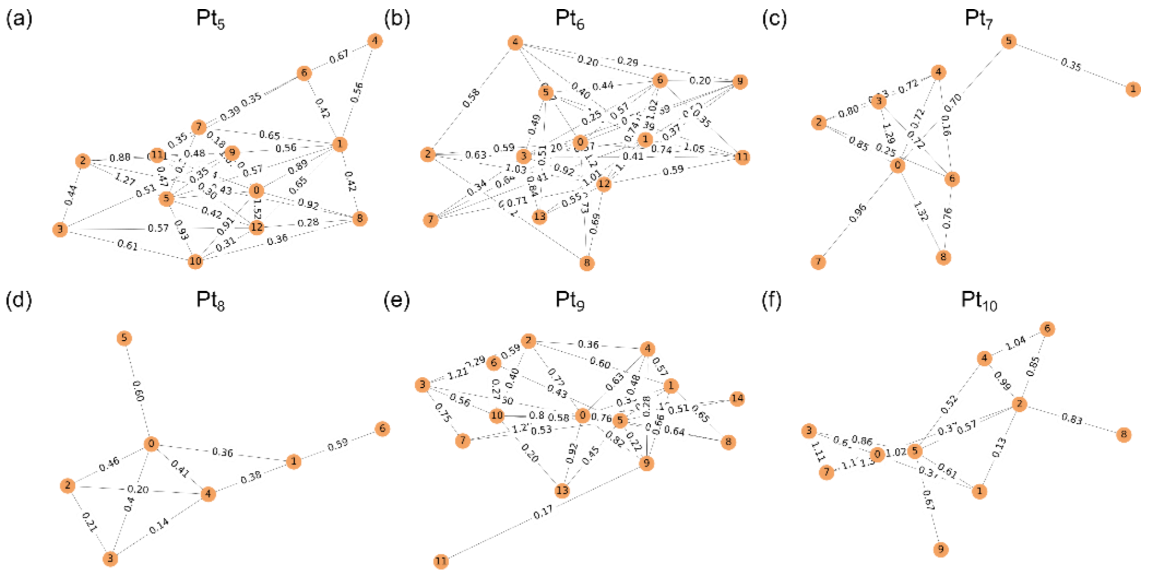

Supplementary Figure 39. The isomer networks over different Pt cluster (Pt_5 to Pt_{10}) supported on TiO_2. Isomerization pathways are represented as graph networks where nodes denote specific structural isomers and edges indicate elementary transformation steps. Numerical labels on edges quantify the forward activation barriers from stable to metastable configurations, expressed in eV.

4. Supplementary Tables

Supplementary Table 1. Structural and topological properties of copper clusters (Cu_2–Cu_{10}). The table summarizes key parameters, including the apparent barrier energy (E_a, eV), the second difference in time (C, s), the number of structural isomers (i), and the count of possible transition networks (N) for each cluster size.

Species	E_a (eV)	C (log (t, s))	Isomers (i)	Networks (N)
Cu_2	0.26	–12.20	2	1
Cu_3	0.33	–12.36	11	18
Cu_4	0.33	–11.39	11	20
Cu_5	0.59	–11.98	16	28
Cu_6	0.34	–12.24	22	75
Cu_7	0.52	–11.74	17	30
Cu_8	0.28	–13.30	13	42
Cu_9	0.89	–11.53	8	8
Cu_{10}	0.70	–12.39	14	32

Supplementary Table 2. CO desorption energetics of the Cu clusters (Cu_2–Cu_{10}). The table summarizes the average CO desorption energy ($E_{des\ ave}$, eV), as well as the maximum ($E_{des,max}$, eV) and minimum ($E_{des,min}$, eV) desorption energies for each cluster size, reflecting the variation in CO binding strength across different adsorption configurations.

Species	$E_{des,\ ave}$ (eV)	$E_{des,\ max}$ (eV)	$E_{des,\ min}$ (eV)
Cu_2	1.14	1.36	0.91
Cu_3	0.90	1.03	0.77
Cu_4	0.90	0.98	0.84
Cu_5	1.00	1.49	0.65
Cu_6	0.86	1.55	0.03
Cu_7	0.91	1.53	0.55
Cu_8	0.87	1.23	0.63
Cu_9	0.72	1.16	0.56
Cu_{10}	0.73	0.94	0.50

Supplementary Table 3. Statistical distribution of CO residence times on Cu clusters (Cu_2–Cu_{10}) at 300 K. K. The table summarizes the logarithms of the average residence time $\log_{10}(\tau_{ave}, s)$, maximum residence time $\log_{10}(\tau_{max}, s)$, and minimum residence time $\log_{10}(\tau_{min}, s)$ for each cluster. The results highlight the decreasing CO residence time on larger clusters, indicating enhanced desorption dynamics with increasing cluster size.

Species	$\log_{10}(\tau_{ave}, s)$	$\log_{10}(\tau_{max}, s)$	$\log_{10}(\tau_{min}, s)$
Cu_2	3.34	6.35	–0.52
Cu_3	–0.69	0.81	–2.97
Cu_4	–0.69	–0.03	–1.79
Cu_5	–2.76	0.03	–5.85
Cu_6	–2.32	8.77	–16.27
Cu_7	–1.48	–0.33	–7.53
Cu_8	–2.16	–6.57	–6.19
Cu_9	–4.68	–6.73	–7.37
Cu_{10}	–4.51	–9.39	–8.37

s

Supplementary Table 4. Statistical distribution of CO desorption rate on Cu clusters (Cu_2–Cu_{10}) at 300 K. The table summarizes the logarithms of the CO desorption rate in the N_C, $\log_{10}(r_{struct}, s^{-1})$, and in the couple process, $\log_{10}(r_{NC}, s^{-1})$ for each cluster.

Species	$\log(r_{struct})$	$\log(r_{Nc})$	$\log(r_{Nc}/r)$
Cu_2	1.86	1.86	0.00
Cu_3	−2.12	−2.12	0.00
Cu_4	−4.45	−4.45	0.00
Cu_5	3.25	4.19	0.96
Cu_6	2.67	4.54	1.87
Cu_7	0.13	3.33	3.20
Cu_8	1.84	3.33	1.49
Cu_9	5.17	4.28	−0.89
Cu_{10}	5.84	5.50	−0.33

Supplementary Table 5. Logarithmic time to thermodynamic stabilization log(*t*, s) for the number of metal clusters (5–10 atoms) at 300 K. The table lists the stabilization times for the Cu, Ag, Au, Ru, Rh, Pd, and Pt clusters, revealing distinct kinetic behaviors across different metals and cluster sizes.

Numbers of atoms	Cu	Ag	Au	Ru	Rh	Pd	Pt
5	–6.23	3.20	–7.06	–3.92	–4.76	–9.88	–7.83
6	–8.90	–7.73	–10.16	/	–8.22	–7.46	–8.30
7	–6.70	–9.72	–7.69	–3.75	–3.77	–7.95	–5.98
8	–11.50	–8.77	–6.49	–6.82	–5.78	–10.07	–9.47
9	3.81	–8.48	–5.06	2.08	–8.49	–8.71	–8.98
10	2.79	–11.42	–7.22	–4.14	–10.28	–8.00	–7.65

Supplementary Table 6. Range of structural reconstruction energy barriers (ΔE_{recon}) across metal clusters (5–10 atoms). This table reports the energy span between the highest and lowest reconstruction barriers (in eV) for each cluster size and metal type. These values reflect the degree of structural heterogeneity and kinetic accessibility of metastable states, which vary markedly with both cluster identity and metal identity.

Numbers of atoms	Cu	Ag	Au	Ru	Rh	Pd	Pt
5	0.93	1.18	0.81	0.33	0.75	0.45	1.38
6	1.01	0.53	1.18	1.18	0.21	0.55	1.07
7	0.87	0.47	0.76	1.16	0.60	0.71	1.20
8	0.68	0.72	0.36	1.36	1.14	0.69	0.46
9	0.69	0.54	0.75	1.07	2.06	1.02	1.11
10	0.93	0.28	0.92	1.38	1.18	0.89	1.21

Supplementary Table 7. Correlations between structural diversity and Tamman temperature across metal clusters. The table summarizes the average number of stable isomers, reconstructed networks, and bulk Tamman temperature (T_m, K) for the metal clusters (Cu, Ag, Au, Ru, Rh, Pd, and Pt).[25]

Parameters	Cu	Ag	Au	Ru	Rh	Pd	Pt
Isomers	18.00	11.17	15.33	6.67	6.83	10.00	11.00
Networks	35.83	17.67	19.83	8.83	10.33	14.67	26.00
T_m	678	617	668	1,362	1,129	914	1,014

Supplementary Table 8. CO desorption energies of metal clusters (Cu, Ag, Au, Ru, Rh, Pd, and Pt) with various numbers of clusters (5–10 atoms). The table lists the average adsorption energy of CO (in eV) as a function of cluster size and metal identity.

Numbers of atoms	Cu	Ag	Au	Ru	Rh	Pd	Pt
5	1.01	0.33	0.91	1.49	1.61	1.35	1.69
6	0.86	0.54	0.75	1.64	1.34	1.6	1.86
7	0.91	0.33	0.66	1.62	1.97	1.58	1.15
8	0.87	0.44	0.5	1.26	1.52	1.27	0.87
9	0.72	0.3	0.44	1.7	1.61	1.89	1.25
10	0.73	0.27	0.39	1.43	1.92	2.23	1.47

Supplementary Table 9. Rate of CO adsorption/desorption across metal clusters (5–10 atoms) at 300 K and 1 bar. The table summarizes the rate constants (log(*r*)) for CO adsorption/desorption processes on clusters composed of seven transition metals (Cu, Ag, Au, Ru, Rh, Pd, and Pt). Negative values indicate slower kinetics, reflecting stronger CO binding or sluggish structural adaptation, whereas positive values correspond to faster CO turnover.

Numbers of atoms	Cu	Ag	Au	Ru	Rh	Pd	Pt
5	–0.67	10.75	1.01	–8.74	–10.75	–6.39	–12.10
6	1.85	7.22	3.0	–11.29	–6.22	–10.59	–14.95
7	1.01	10.75	5.21	–10.92	–16.80	–10.25	–3.02
8	1.68	8.91	7.90	–4.87	–9.24	–5.04	1.68
9	4.20	11.26	8.91	–12.27	–10.75	–15.46	–4.71
10	4.03	11.76	9.75	–7.73	–15.96	–21.17	–8.40

Supplementary Table 10. $\log_{10}(N_c)$ across metal clusters (5–10 atoms) at 300 K. The table reports the N_c for clusters of seven transition metals. The N_c reflects the coupling level of structural reconstruction and reactions in each metal system.

Numbers of atoms	Cu	Ag	Au	Ru	Rh	Pd	Pt
5	–0.53	16.72	0.62	–2.10	–4.14	–6.64	–8.02
6	0.72	3.68	–0.87	/	–4.88	–6.75	–10.21
7	–0.98	3.80	2.50	–3.23	–6.78	–7.03	–0.73
8	–2.42	3.64	5.32	–2.68	–4.26	–6.03	–1.39
9	6.32	5.34	7.35	1.79	–7.87	–10.92	–4.74
10	2.79	2.71	5.69	–1.71	–12.79	–13.63	–5.62

Supplementary Table 11. CO desorption energies (in eV) as a function of surface coverage for 5-atom metal clusters. The table summarizes the evolution of CO desorption energy on Ru_5, Rh_5, Pd_5, and Pt_5 clusters with increasing CO coverage (0 to 1.0 ML). The coverage-dependent variation reflects the balance between adsorbate–metal interactions and structural reorganization, with distinct behaviors observed across different metals.

Coverage	Ru_5	Rh_5	Pd_5	Pt_5
0	1.79	1.65	1.42	1.99
0.2	1.46	2.45	2.13	1.68
0.4	1.86	2.24	1.34	1.70
0.6	1.66	1.67	1.71	1.46
0.8	0.91	1.37	1.04	1.56
1	1.79	1.65	1.42	1.99

Supplementary Table 12. N_c as a function of surface coverage for 5-atom metal clusters at 300 K. The table summarizes the evolution of N_c on Ru_5, Rh_5, Pd_5, and Pt_5 clusters with increasing CO coverage (0 to 1.0 ML).

Coverage	Ru_5	Rh_5	Pd_5	Pt_5
0	$\sim10^{-22}$	$\sim10^{-12}$	$\sim10^{-14}$	$\sim10^{-7}$
0.2	$\sim10^{-2}$	$\sim10^{-35}$	$\sim10^{-24}$	$\sim10^{-7}$
0.4	$\sim10^{-9}$	$\sim10^{-25}$	$\sim10^{-8}$	$\sim10^{-6}$
0.6	$\sim10^{-2}$	$\sim10^{-6}$	$\sim10^{-14}$	$\sim10^{-14}$
0.8	$\sim10^{5}$	$\sim10^{14}$	$\sim10^{2}$	$\sim10^{-5}$
1	$\sim10^{-4}$	$\sim10^{-5}$	$\sim10^{-6}$	$\sim10^{-10}$

Supplementary Table 13. Results for Cu_5 with and without oxygen vacancies at 300 K. The table summarizes the results for Cu_5/TiO_2, reflecting the differences with and without oxygen vacancies. N represents the network connectivity, and i is the number of isomers. $E_{a,recon}$ and E_{des} are the barrier energies of the reconstruction and desorption energies, respectively. N_c is the cycle count. r is the overall rate of CO adsorption/desorption.

Results	N_c with oxygen vacancy	N_c without oxygen vacancy
N	19	32
i	18	18
$E_{a,recon}$ (eV)	0.57	0.46
E_{des} (eV)	0.98	1.00
N_c	~1	$\sim 10^{-2}$
r	10^{-7}	10^{-8}

Supplementary Table 14. Results for Cu_5 with different supports at 300 K. The table summarizes the results for Cu_5 supported on different metal oxidations. N represents the network connectivity, and i is the number of isomers. $E_{a,recon}$ is the barrier energy of the reconstruction. N_c is the cycle count.

Parameters	MgO	TiO_2
i	23	18
N	79	32
$E_{a,recon}$ (eV)	0.39	0.46
N_c	$\sim 10^{-4}$	$\sim 10^{-2}$

Supplementary Table 15. Number of networks and equilibrium times of the structures (τ_{struct}) with different RMSDs of the Cu_5 and Cu_9 clusters. The left and right represent the Cu_5 and Cu_9 clusters, respectively.

RMSD (Å)	Number of Networks	$\log_{10}(\tau_{struct}$ / s)
4.0	28/9	−2.26/3.26
4.5	35/10	−4.01/3.26
5.0	44/13	−4.04/1.18

5. Supplementary References

1. Vilhelmsen LB, Hammer B. A genetic algorithm for first principles global structure optimization of supported nano structures. *J Chem Phys*. 2014; **141**(4): 044711. doi: 10.1063/1.4886337
2. Vilhelmsen LB, Hammer B. Systematic Study of Au-6 to Au-12 Gold Clusters on MgO(100) F Centers Using Density-Functional Theory. *Phys Rev Lett*. 2012; **108**(12): 126101. doi: 10.1103/physrevlett.108.126101
3. Ronne N, Christiansen MPV, Slavensky AM *et al.* Atomistic structure search using local surrogate model. *J Chem Phys*. 2022; **157**(17). doi: 10.1063/5.0121748
4. Jensen PB, Lysgaard S, Quaade UJ *et al.* Designing mixed metal halide ammines for ammonia storage using density functional theory and genetic algorithms. *Phys Chem Chem Phys*. 2014; **16**(36): 19732-19740. doi: 10.1039/c4cp03133d
5. Mantere T, Alander JT. Evolutionary software engineering, a review. *Appl Soft Comput*. 2005; **5**(3): 315-331. doi: 10.1016/j.asoc.2004.08.004
6. Deaven DM, Ho KM. Molecular-Geometry Optimization with a Genetic Algorithm. *Phys Rev Lett*. 1995; **75**(2): 288-291. doi: 10.1103/physrevlett.75.288
7. Deaven DM, Tit N, Morris JR *et al.* Structural optimization of Lennard-Jones clusters by a genetic algorithm. *Chem Phys Lett*. 1996; **256**(1-2): 195-200. doi: 10.1016/0009-2614(96)00406-x
8. Zhu Q, Oganov AR, Glass CW *et al.* Constrained evolutionary algorithm for structure prediction of molecular crystals: methodology and applications. *Acta Crystallogr B*. 2012; **68**: 215-226. doi: 10.1107/s0108768112017466
9. Sun G, Sautet P. Active Site Fluxional Restructuring as a New Paradigm in Triggering Reaction Activity for Nanocluster Catalysis. *Acc Chem Res*. 2021; **54**(24): 4595-4595. doi: 10.1021/acs.accounts.1c00413
10. Poths P, Alexandrova AN. Theoretical Perspective on Operando Spectroscopy of Fluxional Nanocatalysts. *J Phys Chem Lett*. 2022; **13**(19): 4321-4334. doi: 10.1021/acs.jpclett.2c00628
11. Pineda M, Stamatakis M. Kinetic Monte Carlo simulations for heterogeneous catalysis: Fundamentals, current status, and challenges. *J Chem Phys*. 2022; **156**(12). doi: 10.1063/5.0083251
12. Gillespie DT. Exact stochastic simulation of coupled chemical reactions. *J Phys Chem*. 1977; **81**(25): 2340-2361. doi: 10.1021/j100540a008
13. Thomas N, Smidt T, Kearnes S *et al.* Tensor field networks: Rotation-and translation-equivariant neural networks for 3d point clouds. *arXiv preprint arXiv:180208219*. 2018.

14. Fiorin G, Klein ML, Hénin J. Using collective variables to drive molecular dynamics simulations. *Mol Phys*. 2013; **111**(22-23): 3345-3362. doi: 10.1080/00268976.2013.813594

15. Thompson AP, Aktulga HM, Berger R *et al.* LAMMPS-a flexible simulation tool for particle-based materials modeling at the atomic, meso, and continuum scales. *Comput Phys Commun*. 2022; **271**: 108171. doi: 10.1016/j.cpc.2021.108171
16. Henin J, Fiorin G, Chipot C *et al.* Exploring multidimensional free energy landscapes using time-dependent biases on collective variables. *J Chem Theory Comput*. 2010; **6**(1): 35-47. doi: 10.1021/ct9004432
17. Darve E, Rodríguez-Gómez D, Pohorille A. Adaptive biasing force method for scalar and vector free energy calculations. *J Chem Phys*. 2008; **128**(14). doi: 10.1063/1.2829861
18. Lesage A, Lelièvre T, Stoltz G *et al.* Smoothed Biasing Forces Yield Unbiased Free Energies with the Extended-System Adaptive Biasing Force Method. *The Journal of Physical Chemistry B*. 2017; **121**(15): 3676-3685. doi: 10.1021/acs.jpcb.6b10055
19. Barducci A, Bonomi M, Parrinello M. Metadynamics. *Wiley Interdiscip Rev: Comput Mol Sci*. 2011; **1**(5): 826-843. doi: 10.1002/wcms.31
20. Xie W, Xu J, Chen J *et al.* Achieving Theory–Experiment Parity for Activity and Selectivity in Heterogeneous Catalysis Using Microkinetic Modeling. *Acc Chem Res*. 2022; **55**(9): 1237-1248. doi: 10.1021/acs.accounts.2c00058
21. Zientara M, Jakubczyk D, Litniewski M *et al.* Transport of Mass at the Nanoscale during Evaporation of Droplets: the Hertz–Knudsen Equation at the Nanoscale. *J Phys Chem C*. 2013; **117**(2): 1146-1150. doi: 10.1021/jp3091478
22. Zhang YL, Hu C, Jiang B. Embedded Atom Neural Network Potentials: Efficient and Accurate Machine Learning with a Physically Inspired Representation. *J Phys Chem Lett*. 2019; **10**(17): 4962-4967. doi: 10.1021/acs.jpclett.9b02037
23. De S, Bartók AP, Csányi G *et al.* Comparing molecules and solids across structural and alchemical space. *Phys Chem Chem Phys*. 2016; **18**(20): 13754-13769. doi: 10.1039/c6cp00415f
24. Hagberg A, Swart PJ, Schult DA. *Exploring network structure, dynamics, and function using NetworkX*. Los Alamos National Laboratory (LANL); 2007 (DHHS publication no.: Report Number)| (GPO o. Document Number)|.
25. Argyle MD, Bartholomew CH. Heterogeneous Catalyst Deactivation and Regeneration: A Review. *Catalysts*. 2015; **5**(1): 145-269. doi: 10.3390/catal5010145
